# Processing of progranulin into granulins involves multiple lysosomal proteases and is affected in frontotemporal lobar degeneration

**DOI:** 10.1186/s13024-021-00472-1

**Published:** 2021-08-03

**Authors:** Swetha Mohan, Paul J. Sampognaro, Andrea R. Argouarch, Jason C. Maynard, Mackenzie Welch, Anand Patwardhan, Emma C. Courtney, Jiasheng Zhang, Amanda Mason, Kathy H. Li, Eric J. Huang, William W. Seeley, Bruce L. Miller, Alma Burlingame, Mathew P. Jacobson, Aimee W. Kao

**Affiliations:** 1 Department of Neurology, Memory and Aging Center, University of California, San Francisco, California 94143 USA; 2 Department of Pharmaceutical Chemistry, University of California, San Francisco, California 94143 USA; 3 Department of Pathology, University of California, San Francisco, California 94143 USA

**Keywords:** Progranulin, Granulin, Frontotemporal lobar degeneration, Lysosome, Protease, pH, Asparagine endopeptidase

## Abstract

**Background:**

Progranulin loss-of-function mutations are linked to frontotemporal lobar degeneration with TDP-43 positive inclusions (FTLD-TDP-*Pgrn*). Progranulin (PGRN) is an intracellular and secreted pro-protein that is proteolytically cleaved into individual granulin peptides, which are increasingly thought to contribute to FTLD-TDP-*Pgrn* disease pathophysiology. Intracellular PGRN is processed into granulins in the endo-lysosomal compartments. Therefore, to better understand the conversion of intracellular PGRN into granulins, we systematically tested the ability of different classes of endo-lysosomal proteases to process PGRN at a range of pH setpoints.

**Results:**

In vitro cleavage assays identified multiple enzymes that can process human PGRN into multi- and single-granulin fragments in a pH-dependent manner. We confirmed the role of cathepsin B and cathepsin L in PGRN processing and showed that these and several previously unidentified lysosomal proteases (cathepsins E, G, K, S and V) are able to process PGRN in distinctive, pH-dependent manners. In addition, we have demonstrated a new role for asparagine endopeptidase (AEP) in processing PGRN, with AEP having the unique ability to liberate granulin F from the pro-protein. Brain tissue from individuals with FTLD-TDP-*Pgrn* showed increased PGRN processing to granulin F and increased AEP activity in degenerating brain regions but not in regions unaffected by disease.

**Conclusions:**

This study demonstrates that multiple lysosomal proteases may work in concert to liberate multi-granulin fragments and granulins. It also implicates both AEP and granulin F in the neurobiology of FTLD-TDP-*Pgrn*. Modulating progranulin cleavage and granulin production may represent therapeutic strategies for FTLD-*Pgrn* and other progranulin-related diseases.

**Supplementary Information:**

The online version contains supplementary material available at 10.1186/s13024-021-00472-1.

## Background

Progranulin (PGRN) is an evolutionarily conserved glycoprotein with functions in inflammation, wound healing, tumorigenesis, and neuroprotection [1, 2]. Dosage of PGRN plays a central role in its cellular functions as haploinsufficiency of PGRN leads to an adult-onset neurodegenerative disorder, frontotemporal lobar degeneration (FTLD-TDP-*Pgrn*), while complete loss of PGRN leads to a childhood lysosomal storage disorder called neuronal ceroid lipofuscinosis (NCL) [3–5]. As a molecule with complex layers of regulation, PGRN can also be proteolytically cleaved into multiple biologically active, disulfide-rich peptides known as granulins which, in some instances, have contrasting functions to its precursor [6–10]. Although granulins were identified before PGRN, the regulation of granulin production remains poorly understood [11]. Since haploinsufficiency of PGRN protein could potentially also affect granulin levels, understanding granulin production may shed light on how partial loss of PGRN causes age-associated neurodegeneration.

**Fig. 1:**
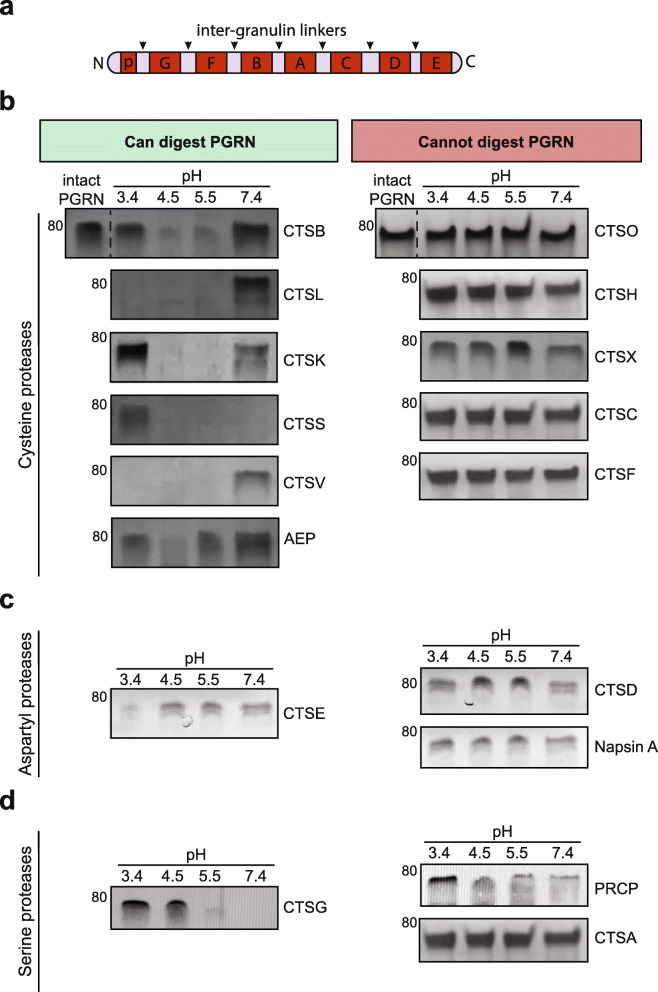
Progranulin can be digested by multiple lysosomal proteases in vitro*.* **a**, Schematic representation of progranulin (PGRN) protein. Black arrows indicate inter-granulin linkers, individual granulins named granulins A-G (~ 7 kD each) and paragranulin (p) (~ 3.5 kD) are annotated. **b**-**d**, 1 μM of each enzyme was incubated with 400 ng of recombinant human PGRN at the indicated pH for 20 min. The proteins were silver stained (shown in greyscale) to assess the presence or absence of PGRN at every condition. All assays were repeated *n* = 3 times

Proteolytic processing of PGRN liberates up to eight granulin peptides, named paragranulin (~ 3.5kD) and granulins A through G (~7kD each) (Fig. 1a). Fragments consisting of multiple granulins have also been previously described [12, 13], and these multi-granulin fragments (MGFs) exert biological activities as well [14]. PGRN is secreted into the extracellular matrix where proteases, such as neutrophil elastase, may process PGRN [6, 8, 15]. Intracellularly, PGRN localizes to endo-lysosomes and recent studies have shown that intracellular processing of PGRN into granulins also occurs in the endo-lysosomal compartments [16–20]. The lysosomal cysteine proteases, cathepsins B (CTSB) and L (CTSL), were identified as PGRN proteases in human and mouse models [17, 19, 20]. However, a comprehensive study of intracellular PGRN proteases has not yet been performed.

To better understand the intracellular processing of PGRN into granulins, we set out to catalog the endo-lysosomal proteases that regulate PGRN processing into granulins. Using in vitro protease cleavage assays, we identified multiple lysosomal proteases that can process PGRN to multi-granulin fragments and individual granulins. Interestingly, they did so in a pH-dependent manner that is distinctive depending on the protease. In a neuronal cell model, we showed that asparagine endopeptidase (AEP, also known as legumain) is a novel PGRN protease that can specifically liberate granulin F. Finally, FTLD-TDP-*Pgrn* subjects exhibit relatively higher AEP activity and correlated higher granulin F levels in diseased middle frontal gyrus (MFG) compared to non-diseased inferior occipital cortex (IOC) regions of the brain. This study is the first to systematically identify a suite of endo-lysosomal proteases that likely work in concert to process PGRN to granulins and reveals AEP as a new PGRN protease with relevance in FTLD-TDP. It also reveals the bioactive MGFs and granulins that can be produced by each protease, which could become important in the development of FTLD-*Pgrn* therapies targeted at preventing PGRN cleavage.

## Methods

### In vitro cleavage assay

For in vitro cleavage assays, 400 ng of recombinant human progranulin (R&D #CF-2420) was incubated with or without 1 μM of each protease. Depending on the pH setpoint, the following buffers were used: 100 mM sodium citrate pH 3.4, 50 mM sodium acetate pH 4.5 or 5.5, 50 mM 2-(N-morpholino)-ethanesulfonic acid (MES) buffer pH 6.5, 100 mM phosphate buffer saline (PBS) pH 7.4 with 1 mM EDTA and 2 mM DTT for the time indicated at 37 °C water bath. The cleavage was performed in a total volume to 19.5 μl. Protease activity was stopped by adding 7.5 μl of NuPAGE 4X LDS (Fisher Scientific #NP0007), 3 μl of 10X reducing agent (i.e., 50 μM) (Fisher #NP0009) and denatured for 10 min at 70 °C. Of note, we used these denaturing conditions to reduce the potential for dimerization of fragments (a known possibility with PGRN) [21].

For the time-course assays, 400 ng of recombinant human progranulin (R&D #CF-2420) was incubated with 50 nM or 1uM of the enzyme indicated in the same reaction conditions as mentioned above. The reaction was allowed to proceed for the time-point indicated. The reaction was stopped at mentioned above.

All samples were run on precast NOVEX 4–12% Bis-Tris gels (Fisher #NP0321PK2) using MES buffer (Fisher #NP0002). The gel was then either fixed in 40% ethanol and 10% acetic acid for silver and Coomassie staining or transferred onto nitrocellulose membranes for western blotting analysis.

#### Silver stain

Silver staining was performed according to manufacturer’s instructions with SilverQuest silver staining kit (Thermo Fisher #LC6070).

#### Coomassie stain

Coomassie staining was done using BIO-RAD QC colloidal Coomassie stain (Bio-rad #160–0803). The gel was stained overnight and was removed by washing in milli-Q water for 3 h.

#### Recombinant proteases

Cathepsin E (R&D #1294-AS), Cathepsin D (R&D #1014-AS), Napsin A (R&D #8489-NA), Cathepsin G (Millipore #219873), Cathepsin A (R&D #1049-SE), Pro-X-carboxypeptidase (R&D #7164-SE-010), Cathepsin L (Millipore #219402), Cathepsin B (Millipore #219364), Cathepsin K (Millipore #219461), Cathepsin S (R&D #1183-CY), Cathepsin V (R&D #1080-CY), Asparagine endopeptidase/legumain (R&D #2199-CY), Cathepsin H (R&D #7516-CY-010), Cathepsin C (R&D #1071-CY), Cathepsin O (Abcam #ab267932), Cathepsin F (Abcam #ab240858), and Cathepsin X (R&D #934-CY).

#### Protease activation and confirmation of activity

Cathepsins E, D, G, L, B, K, S, V, F, and O as well as Asparagine endopeptidase, Napsin A, and Pro-X-carboxypeptidase require no activation, according to the vendor, and were used as purchased. Cathepsin A, C, and H require pre-activation before use. For cathepsin A and cathepsin C activation, 1 μM of each protease was incubated with 200 nM of cathepsin L at room temperature for 1 h. After 1 h, 50 μM of benzyloxycarbonyl FY(*t*-Bu)-DMK, an irreversible, highly specific inhibitor of cathepsin L (Sigma #219427) was added to quench cathepsin L activity. Once activated, cathepsin A and C were used in the experiments outlined. Similarly, for cathepsin H activation, 1 μM of cathepsin H was incubated with 500 nM of Thermolysin (R&D #3097-ZN) at room temperature for 3 h. After 3 h, 1 mM of Phosphoramidon (Tocris Bioscience #6333), a specific thermolysin inhibitor, was added to quench Thermolysin activity. Once activated, cathepsin H was used in the experiments outlined. To confirm each protease was enzymatically active, protease activity control experiments were performed using a FRET-based fluorogenically labeled casein assay (Biovision #K781), according to the vendor’s protocol. In brief, 1 μM of each protease was added to a 50 μL reaction mixture using the recommended concentration of control fluorogenic substrate in a buffer consisting of 50 mM sodium acetate buffer at pH 4.5 and in the presence of 1 mM EDTA and 2 mM DTT. The assay was performed at 37 °C for 60 min and readings were taken every 2 min using Tecan Infinite M Plex plate reader.

### Statistical analysis

Details of the statistical test used for each experiment is in figure legends along with n and *p* value. All data is represented as mean ± SD. Statistical analysis was performed using GraphPad Prism 8 (GraphPad Software, La Jolla, California USA). A *p-*value < 0.05 was considered significant.

### Cell culture, treatment, and lysis

#### Cell culture

SH-SY5Y human neuroblastoma cells were obtained from ATCC (CRL-2266) and maintained 1:1 EMEM/F12 media (ATCC #30–2003/Thermo #11765062) supplemented with 10% FBS and 1% Penicillin-Streptomycin (Thermo Fisher #15140122). SH-SY5Y cells were differentiated by treating with 10 μM all-trans retinoic acid (Sigma #R2625) for 6 days in EMEM/F12 media supplemented with 10% FBS and 1% Penicillin-Streptomycin, followed by 4 days of treatment with 50 ng/mL of BDNF (Peprotech #450-02B) in EMEM/F12 media supplemented with only 1% Penicillin-Streptomycin. HEK293FT were maintained in high glucose DMEM media with 10% FBS and 1% Penicillin-Streptomycin. The WTC11 and isogenic progranulin KO iPSC cell lines [22, 23] were gifted by Bruce Conklin’s lab (Gladstone Institute, UCSF). For routine culture, iPSCs were plated in hESC matrigel (Corning #354277) coated plate with mTeSR plus (Stemcell Technologies #05825) and ROCKi (Stemcell Technologies #72304). iPSCs were maintained in mTeSR plus until confluent for lysate collection. Cells were washed with PBS and dissociated with Accutase (Thermo Fisher Scientific #A1110501).

#### Cell treatments

For siRNA studies, day 10 differentiated SHSY5Y cells had their media replaced with Accell siRNA Delivery Media (Dharmacon #B-005000-500) supplemented with 50 ng/ml BDNF (Peprotech #450-02B). Either Accell Non-targeting Pool siRNA (Dharmacon #D-001910-10-20) or Accell Human pooled LGMN siRNA (Dharmacon #E-005924-00-0010) was added to the cells at a concentration of 2uM. After 72 h, the media was replaced with EMEM/F12 with 50 ng/mL BDNF. Cells were collected after 96 h and collected for western blot analysis. For overexpression studies, pCMV6 mammalian expressing vectors carrying the cDNA of AEP and CTSL were ordered from Origene (#RC224975 and #RC203143). XtremeGene HP (Sigma, #06366236001) was used to transiently transfect HEK293FT cells for 24 h following the manufacturers suggestions. The cells were then pelleted and prepared for western blotting as described below.

#### Cell lysis

The cells were collected post-treatment and prepared for western blot analysis. Cells were washed once with PBS, trypsinized, and pelleted. The pellets were lysed in RIPA buffer (Fisher #89900) with protease and phosphatase inhibitors (Sigma #4693124001, #4906837001) and centrifuged at 15000 rpm at 4 °C for 20 min. The supernatant containing the soluble proteins were transferred into a new tube. Estimated protein concentration with a Peirce BCA (Fisher #PI23225) protein assay kit following manufacturer’s instructions. The nitrocellulose membrane was blocked with 5% milk (Fisher #NC9121673) or Odyssey buffer (Li-cor #927–50,010) for 1-2 h. The membranes were cut between 60-50kD and between 20-15kD to obtain PGRN and granulin-specific bands respectively and incubated with primary antibodies overnight [19]. Li-cor secondary antibodies at 1:5000 dilution was used and imaged using Odyssey CLx imager. Western blots were quantified using FIJI software.

### Antibodies

#### Antibody generation

The following epitopes were used to generate the rabbit polyclonal anti-bodies: paragranulin (p-Gran) -TRCPDGQFCPVACCLDPGGASYSCCRPLLD, granulin F (Gran-F) - QCPDSQFECPDEST, and granulin E (Gran-E) - ECGEGHFCHDNQTCCR.

#### Antibody validation

The sensitivity, specificity, and reproducibility of results of the novel p-Gran, Gran-F, and Gran-E antibodies were validated using a protocol similar to Bordeaux, et al. 2010 [24]. In brief, antibody specificity for each antigen was first measured by dot blot. For these experiments, 2.5 μg of each granulin peptide was pipetted onto a nitrocellulose membrane defined into grids. Each antibody was incubated with the membrane overnight at 1:1000 dilution. Signals from the primary antibodies were amplified using species-specific antisera conjugated with horseradish peroxidase (Cell Signaling Technology #7074) and detected with a chemiluminescent substrate detection system ECL and scanned the images with a densitometer. Following successful confirmation of antibody sensitivity, the specificity was evaluated via a peptide blocking experiment. Recombinant human progranulin was incubated with CTSL, a known PGRN protease, at pH 4.5 for 20 min at 37 °C as previously described. Samples were denatured and western blots were performed for peptide blocking analysis. Each primary antibody (p-Gran, Gran-F, Gran-E) was pre-incubated with its respective granulin peptide for 2 h at room temperature with gentle mixing. Optimal primary antibody concentration was incubated with two different peptide amounts and on its own. Primary antibody to peptide ratio was determined by molarity at 10X, 100X, and no peptide. Following incubation, solutions were used to blot membranes overnight at 4 °C. Normal western blot procedure was completed as described.

#### Commercial antibodies

Anti-progranulin C-terminus (Thermo Fisher #40–3400, 1:200), Anti-granulin F (Sigma #HPA008763, 1:250), Anti-AEP (R&D #AF2199, 1:500), anti-Actin (Sigma #MAB1501R, 1:5000), and anti-FLAG (Sigma #F3165).

### Human brain tissue lysis and analysis

Brain tissue was prepared as previously described in Salazar et al., 2015 [12]. Briefly, adjacent tissue blocks were fixed, embedded in paraffin wax, sectioned, stained for hematoxylin and eosin, and rated for astrogliosis (0–3 scale). Absent or low gliosis regions were selected with a score of 0–1, while high levels of gliosis regions with a score of 2–3. From this, we selected the middle frontal gyrus (MFG) with severe degeneration and inferior occipital cortex (IOC) with no degeneration for all assays. The same region was collected from control subjects. Brain tissue samples were weighed, diluted 15-fold with lysis buffer. For WB, samples were lysed in 1X RIPA buffer (Thermo Fisher Scientific #89900), 1% triton-X, (Millipore Sigma #T9284), protease and phosphatase inhibitors. For enzyme activity assays, samples were lysed in PBS (Thermo Fisher #14190250) and 1% triton-X (Millipore Sigma #T9284). Samples were then homogenized for 1 min (pestle pellet motor) and sonicated for 5 rounds of 30 s on and 1 min off (BioRuptor, Diagenode). The lysate was centrifuged at max for 20 min and the soluble supernatant was used for western blot assays. BCA was performed to determine protein concentration according to manufacturer’s instructions. 25-50 μg of total protein from each sample was loaded for western blot analysis. The nitrocellulose membrane was blocked with 5% milk or Odyssey buffer for 1–2 h. The membranes were cut between 60-50kD and between 20-15kD to obtain PGRN and granulin-specific bands respectively and incubated with primary antibodies overnight [19]. Li-cor secondary antibodies at 1:5000 dilution was used and imaged using Odyssey CLx imager. Western blots were quantified using FIJI software.

### qPCR

Differentiated SH-SY5Y cells were pelleted and RNA was extracted using standard phenol:chloroform extraction techniques. 1 μg of RNA was reverse transcribed to cDNA using Superscript III reverse transcription kit following manufacturer’s protocol (Thermo Fisher #18080044), random primers (Thermo Fisher #48190011) and dNTPs (Sigma #11969064001). RT-qPCR was performed using TaqMan Fast Advanced Master Mix (Thermo Fisher #4444557) in an ABI Prism 7900HT Sequence Detection System (Applied Biosystems), with TaqMan FAM probes for human CTSB (Hs00947433_m1), CTSL (Hs009964650_m1), CTSK (Hs00166156_m1), CTSS (Hs00175407_m1), AEP/LGMN (Hs00271599_m1), CTSV (Hs00426731_m1), CTSE (Hs00157213_m1), CTSG (Hs01113415_g1) and the house keeping gene GAPDH (Hs02758991_g1) as a control. Four biological samples were analyzed, each with three technical replicates. mRNA levels of target genes were normalized to the mean of the house keeping gene GAPDH. Data is displayed as relative values compared to GAPDH.

### Enzyme activity assays

#### AEP activity assay

AEP substrate Z-Ala-Ala-Asn-AMC (Bachem #I1865) was used to measure activity. 50 mM sodium acetate buffer pH 5.5 with 2 mM DTT, 1 mM EDTA and 0.1% CHAPS was used as the assay buffer. 100 μM of the substrate was used per reaction. The assay was performed at 37 °C for 60 min and readings were taken every 2 min using Tecan Infinite M Plex plate reader. Activity was measured using excitation wavelength 380 nm and emission wavelength 460 nm. Assays were performed in triplicates for each sample.

#### CTSL activity assay

Biovision CTSL fluorometric assay kit (#K142) was used according to manufacturer’s protocol with some modifications. Since CTSB can also cleave the assay substrate, the assay was performed with 1 mM CTSB inhibitor (CA074_ME, Caymen Chemical Company #18469) to specifically assay CTSL activity. The assay was performed at 37 °C for 60 min and reading were taken every 2 min using a Tecan Infinite M Plex plate reader. Activity was measured using excitation wavelength 400 nm and emission wavelength 505 nm. This assay was performed in triplicates for each sample.

### Identification of AEP cleavage sites

#### In-gel digestion

400 ng of recombinant human progranulin (R&D #CF-2420) was incubated at 37 °C for 60 min with recombinant human AEP (R&D #2199-CY) at pH 4.5. The cleavage was stopped by adding 7.5 μl of NuPAGE 4X LDS (Fisher #NP0007), 3 μl of 10X reducing agent (Fisher #NP0009) and denatured for 10 min at 70 °C. The samples were run on precast NOVEX 4–12% Bis-Tris gels (Fisher #NP0321PK2) using MES buffer (Fisher #NP0002). The gel was fixed, and silver stained as described above. Protein bands were excised and digested in-gel with Endoproteinase Asp-N (Sigma #11054589001) as described previously [25, 26]. The extracted digests were vacuum-evaporated, resuspended in 20 μl of 0.1% formic acid, and desalted using C18 ZipTips (Millipore #ZTC18M096).

#### Mass spectrometry

Asp-N peptides were analyzed by on-line LC-MS/MS using an Orbitrap Fusion Lumos (Thermo Scientific) coupled with a NanoAcquity M-Class UPLC system (Waters, Milford, MA). Peptides were separated over a 15 cm × 75 μm ID 3 μm C18 EASY-Spray column (Thermo Scientific #ES800). Precursor ions were measured from 375 to 1500 m/z in the Orbitrap analyzer (resolution: 120,000; AGC: 4.0e5). Ions charged 2+ to 7+ were isolated in the quadrupole (selection window: 1.6 m/z units; dynamic exclusion window: 30s; MIPS Peptide filter enabled), fragmented by HCD (Normalized Collision Energy: 30%) and measured in the Orbitrap (resolution: 30,000; AGC; 5.0e4). The cycle time was 3 s.

Peaklists were generated using PAVA (UCSF) and searched using Protein Prospector 5.23.0 against the SwissProt database (downloaded 9/6/2016) and a randomized concatenated database. Cleavage specificity was set as Asp-N/AEP (Asn-C) allowing 2 mis-cleavages. Carbamidomethylation of Cys was set as a constant modification and two of the following variable modifications were allowed per peptide: acetylation of protein N-termini, oxidation of Met, oxidation and acetylation of protein N-terminal Met, cyclization of N-terminal Gln, protein N-terminal Met loss, protein N-terminal Met loss and acetylation. Precursor mass tolerance was 20 ppm and fragment mass tolerance was 30 ppm. A subsequent search using the above parameters but limiting the search to the following accession numbers and a user defined PGRN amino acid sequence (the human sequence minus the first 18 amino acids and with a C-terminal 6xHis tag) was used for further analysis: B2FQP3 O08692 P02533 P02666 P02754 P02769 P04264 P28799 P35527 P35908 P85945 Q8PC00 Q91FI1 Q9R4J4 P07711 Q99538. Spectra containing AEP cleavage sites can be viewed in MS-Viewer with the search key “besr1qb6q4” or the following link: http://msviewer.ucsf.edu/prospector/cgi-bin/mssearch.cgi?report_title=MS-Viewer&search_key=besr1qb6q4&search_name=msviewer [27].

## Results

### Multiple lysosomal proteases digest human progranulin in vitro

Previous studies have identified CTSL and CTSB as intracellular PGRN proteases [17, 19], however the endo-lysosomal compartment contains many other proteolytic enzymes that belong to distinctive classes with specific but overlapping peptide recognition motifs [28]. We wondered if other lysosomal enzymes also play a role in processing human PGRN. To identify which proteases can and cannot cleave PGRN, we performed extensive in vitro cleavage studies with commercially available recombinant human lysosomal enzymes. Although we were ultimately interested in which of the proteases can liberate individual granulin peptides, we first asked the question of which lysosomal proteases can *digest* or *break down* the full-length pro-protein in the inter-granulin linker regions (Fig. 1a). Since different proteases have distinct pH setpoints for optimal activity that can be substrate-dependent [28–30], we performed the study across a range of pH settings that represents the stepwise maturation of endo-lysosomal compartments.

Lysosomal proteases are classified based on the amino acid(s) in their active site. We first assayed the largest class of lysosomal proteases, the cysteine protease family [31, 32]. As previously shown [17, 19], the cysteine proteases CTSB and CTSL digested recombinant human PGRN (Fig. 1b). In addition, cathepsins K (CTSK), S (CTSS), V (CTSV) and asparagine endopeptidase (AEP) were also able to digest PGRN within 20 min. At an acidic pH of 4.5 or 5.5, CTSB, CTSL, CTSK, CTSS and CTSV digested most or all of full-length PGRN. AEP only modestly digested PGRN at pH 4.5 and 5.5 within the same time frame. CTSL and CTSV (also known as cathepsin L2), were the only cysteine protease that could digest PGRN at a pH as low as 3.4. Interestingly, CTSS, a lysosomal and secreted enzyme, is the only cysteine protease that can efficiently digest PGRN at both acidic and neutral pH, thereby making it a candidate enzyme to process both intracellular and extracellular PGRN [33]. In contrast, the cysteine cathepsins H (CTSH), C (CTSC), F (CSTF), O (CTSO) and X (CTSX) were unable to digest PGRN at any pH tested (Fig. 1b).

We next tested the aspartic acid family of acid hydrolases, which includes napsin A, cathepsin E (CTSE) and cathepsin D (CTSD). Napsin A, which has been used as a biomarker of human cancers [34, 35], did not degrade PGRN in vitro at any pH tested (Fig. 1c). Cathepsin E (CTSE) is an endo-lysosomal aspartyl protease, highly expressed in immune cells such as microglia [36]. CTSE digested PGRN only at the most acidic pH of 3.4 (Fig. 1c). CTSD has been implicated in multiple neurodegenerative diseases and was previously demonstrated to associate with PGRN, which promotes CTSD maturation and activity [14, 37–43]. One recent study reported that prolonged incubation (16 h) of PGRN with CTSD at pH 3.5 can lead to PGRN cleavage, although low molecular weight granulin-sized bands were not reported [20]. Typically, proteases act on their substrates rapidly, within minutes. To determine if CTSD plays a role in degrading PGRN at more physiological time scales, we incubated mature CTSD with recombinant PGRN for 20 min. Under these in vitro conditions, CTSD did not degrade PGRN at any pH (Fig. 1c). To ensure that CTSD was indeed active, we tested its activity against a known substrate, BSA [43], incubated for 60 min at pH 3.4 and 4.5. Under these conditions, CTSD cleaved BSA but not PGRN (Fig. S1a). Thus, PGRN is likely not a favored CTSD substrate under physiological conditions. We confirmed [20], however, that with prolonged incubation of 16 h, CTSD can digest PGRN (Fig. S1a).

We then tested the final class of lysosomal proteases, the serine proteases. Pro-X-carboxypeptidase (PRCP) and cathepsin A (CTSA) were unable to digest PGRN at any pH tested (Fig. 1d). However, cathepsin G (CTSG), a lysosomal and secreted serine protease digested PGRN at pH 5.5 and 7.4. Therefore, CTSG, like CTSS, is a candidate protease to process both intracellular and extracellular PGRN.

To determine if the enzymes that do not cleave PGRN in our assay (CTSH, CTSC, CTSF, CTSO, CTSX, CTSD, napsin A, PRCP and CTSA) are active against another control substrate, we performed FRET-based cleavage assays with fluorogenically-tagged casein. The results confirm that all enzymes are active at pH 4.5 and can cleave this universal substrate, although with varied efficiencies (Fig. S1b).

Therefore, in total six cysteine (CTSB, CTSL, CTSK, CTSS, CTSV and AEP), one aspartyl (CTSE) and one serine protease (CTSG) rapidly digested full-length PGRN in vitro in a pH-dependent manner (Table 1). Since protease expression can be cell-type specific [28, 44, 45], these results suggest that PGRN processing can be highly regulated, occurring in a protease-specific, pH-dependent manner and possibly both extracellularly and in multiple intracellular compartments.

**Table 1:**
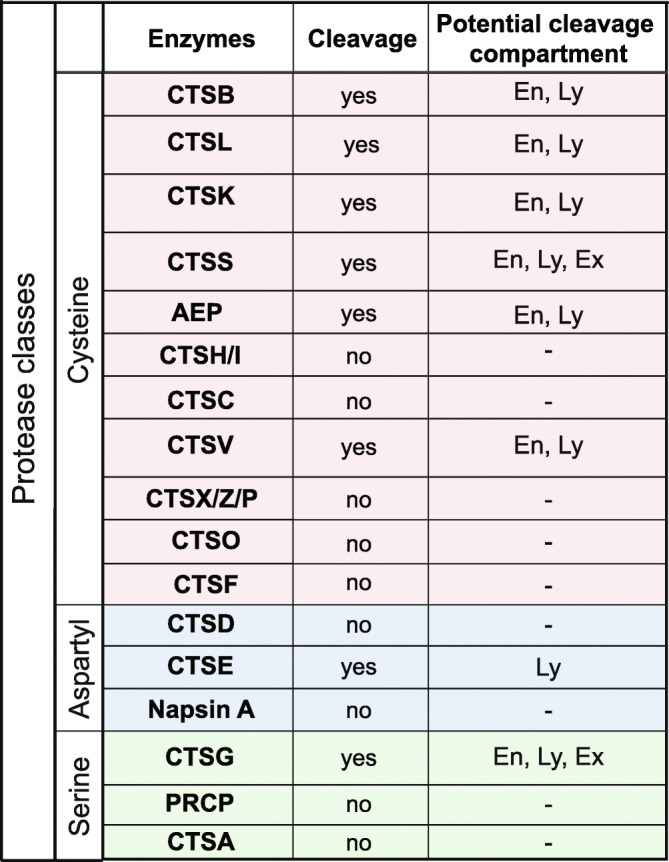
PGRN cleavage by lysosomal proteases in vitro

Summary of whether the enzymes tested could cleave progranulin (PGRN) or not along with the potential compartment it may cleave PGRN depending on the pH it processed progranulin in vitro. En, endosomes; Ly, lysosomes; Ex, extracellular

### PGRN undergoes pH-dependent processing into multi-granulin fragments (MGFs) and individual granulins in vitro

**Fig. 2:**
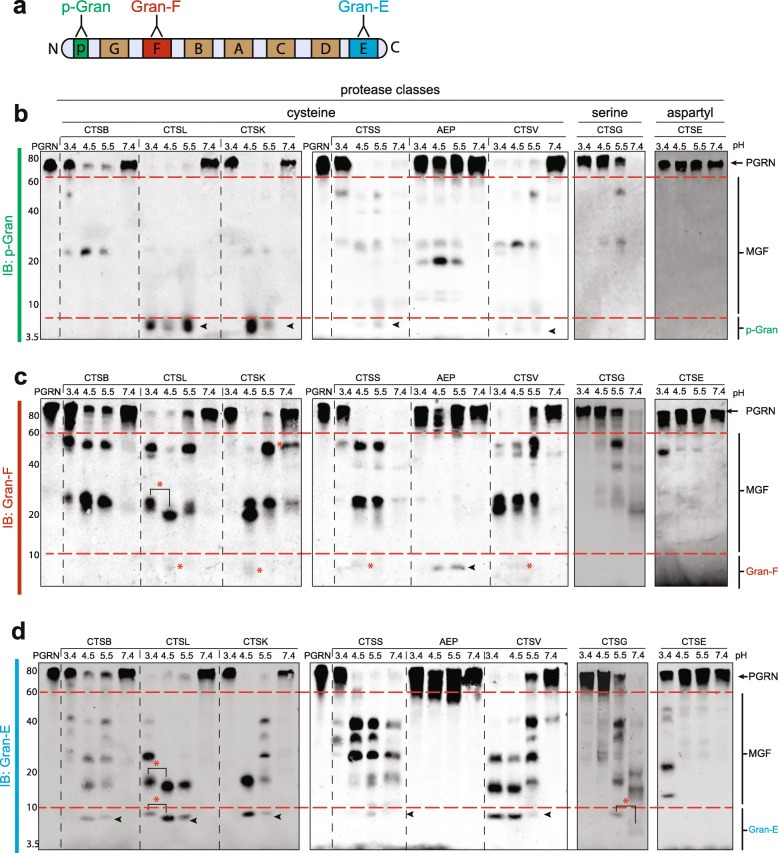
PGRN undergoes pH-dependent processing into multi-granulin fragments and individual granulins in vitro*.* **a**, Schematic representation of the antibodies used. Anti-paragranulin (p-Gran) in green, anti- granulin F (Gran-F) in red and anti-granulin E (Gran-E) in blue. **b-d,** For in vitro protease assays, 400 ng of PGRN was incubated for 20 min with or without 1 μM of each enzyme at the indicated pH. Western blotting analysis was performed using antibodies as indicated. pH-dependent cleavage products are indicated with red asterisks (*). Progranulin (PGRN), multi-granulin fragments (MGFs), cathepsin B (CTSB), cathepsin L (CTSL), cathepsin K (CTSK), cathepsin S (CTSS), asparagine endopeptidase (AEP), cathepsin V (CTSV), cathepsin G (CTSG), cathepsin E (CTSE). A representative of n = 3 replicates is shown

Progranulin is well-known to be a precursor protein for granulins, which are stress-responsive molecules with varying bioactivities [6–10]. Having established a subset of lysosomal proteases that can digest full-length PGRN, we next sought to understand which of these could proteolytically process PGRN into granulin-sized peptides. To do so, we generated custom anti-granulin antibodies raised against the N-terminal paragranulin (p-Gran), a centrally located peptide, granulin F (Gran-F) and C-terminal granulin E (Gran-E) (Fig. 2a and Fig. S2). We then analyzed the results of in vitro cleavage assays for lower molecular weight bands that represent MGFs or individual granulins. Consistent with our previous results, the lysosomal proteases that could not digest PGRN did not release any specific multi-granulin or individual granulin-sized fragments (Fig. S3). Given the large amount of data, we will first discuss the production of the N- and C-terminal granulins, followed by granulin F and finally the MGFs.

The N-terminal paragranulin can be released by a single cleavage after granulin p. Following incubation with CTSL and CTSK, and to a lesser extent in CTSS and CTSV, the anti-p-Gran antibody detected a < 7kD band that likely represents this cleavage event that produces the paragranulin (Fig. 2b and Fig. S4). Similarly, the C-terminal granulin E can be released by a single cleavage after granulin D. Following incubation with CTSL, CTSK, and CTSV, and to a lesser extent CTSB, CTSS and CTSG, the anti-gran-E antibody detected a < 10kD band that likely represents this cleavage and release of Gran-E (Fig. 2d and Fig. S4). Interestingly, the endo-lysosomal aspartyl protease CTSE also had the ability to process PGRN to release an ~12kD peptide containing granulin E, but only at pH 3.4, suggesting that CTSE may have a different cleavage pattern than other proteases (Fig. 2d).

Unlike paragranulin and granulin E, the release of granulin F requires two cleavages (after granulin G and before granulin B.) Most individual proteases were inefficient at cleaving both of these specific linkers. CTSL, CTSK, CTSS and CTV robustly cleaved PGRN into MGFs containing granulin F, but inefficiently released individual granulin F, and only at pH 4.5 (Fig. 2c). AEP was a notable exception, as it could robustly release granulin F at pH 4.5 and 5.5. However, AEP was unable to liberate the N and C-terminal granulins (Fig. 2c and S4). These results suggest that different proteases cleave at different inter-granulin regions in PGRN in a pH-specific manner. This is summarized in Fig. S5.

Given that each intact PGRN molecule contains up to eight granulins and seven inter-granulin linkers, the potential number of different multi-granulin fragments could be quite high (up to 86 potential products). However, when we observed the actual pattern of multi-granulin fragments, we found that their molecular weights were largely similar across both cysteine and serine proteases (Fig. 2b-d). This suggests similar cleavage patterns for the different enzymes, albeit with different abundances. The notable exception to this pattern, once again, was AEP, which exhibited a distinctive pattern of intermediate bands. Interesting differences in MGF production also emerged across pH, including shifts in the size of multi-granulin and individual granulin-sized bands (Fig. 2c-d, red asterisk).

To determine the efficiency of processing PGRN at different inter-granulin domains, we performed time-course experiments for CTSL and CTSB (Fig. S6). Since both these enzymes digest almost all recombinant PGRN within 20 min (Figs. 1b and 2b), we decreased the enzyme concentration by 20-fold to 50 nM and maintained the substrate concentration. Cleavage products were collected at the time points indicated and western blotting performed using anti-p-Gran, Gran-F and Gran-E antibodies. The time-course data suggests that CTSL is highly efficient at releasing paragranulin and Gran-E from PGRN (Fig. S6a). CTSL can cleave the inter-granulin linker between p-G as early as 2.5mins releasing paragranulin and between D-E within 10 min releasing granulin E. In contrast, CTSL does not liberate Gran-F even when incubated up to 60 min at this concentration. This further confirms are results that CTSL can robustly process PGRN to release N-terminus paragranulin and C-terminus granulin E but is unable to liberate granulin F at the same concentration (Fig. 2). Interestingly, at this lower concentration, CTSB, another documented PGRN protease, is unable to process PGRN suggesting a much lower PGRN cleaving efficiency in comparison to CTSL (Fig. S6b). This data suggests that CTSL is more efficient than CTSB at processing PGRN, and primarily releases the N- and C-terminal granulins.

In summary, our results demonstrate that eight lysosomal proteases are capable of digesting full-length human PGRN in vitro. Moreover, these findings demonstrate that each of these enzymes can cleave PGRN into one or more individual granulin (Fig. S5b). When tested in isolation, the majority of cysteine, aspartyl and serine proteases tended to liberate N- and/or C-terminal granulins. In contrast, AEP was unable to liberate the N- and/or C-terminal granulins but robustly liberated granulin F, present in the center of the pro-protein. Furthermore, one cysteine (CTSS) and one serine protease (CTSG) processed PGRN into granulins at neutral pH (7.4) while an aspartyl protease (CTSE) could only process PGRN at highly acidic pH (3.4).

### Asparagine endopeptidase is a PGRN protease that liberates granulin F

To assess the specificity of our custom anti-granulin antibodies in cell lysates, we used a pair of previously published, isogenic wild-type and progranulin knockout (KO) iPSC cell lines [22, 23]. The anti-p-gran, gran-F and gran-E antibodies recognized full-length PGRN from iPSC whole cell lysates, but contained many non-specific bands (Fig. S7a). In contrast, the commercially available Invitrogen and Sigma anti-progranulin antibodies robustly recognize full-length progranulin (Fig. S7b). In regards to PGRN cleavage products, the anti-Gran-F antibody and the Sigma antibody both recognized a granulin-sized band, which according to a recent study of all available commercial PGRN antibodies [19], is likely to be granulin F. With all of the antibodies, comparison of *Pgrn* WT and KO cells revealed many non-specific bands. Given these findings and the limited quantities of the custom antibodies available, we chose to utilize the Invitrogen and the Sigma antibodies to detect full-length endogenous PGRN and granulin F, respectively, in cell-based studies.

**Fig. 3:**
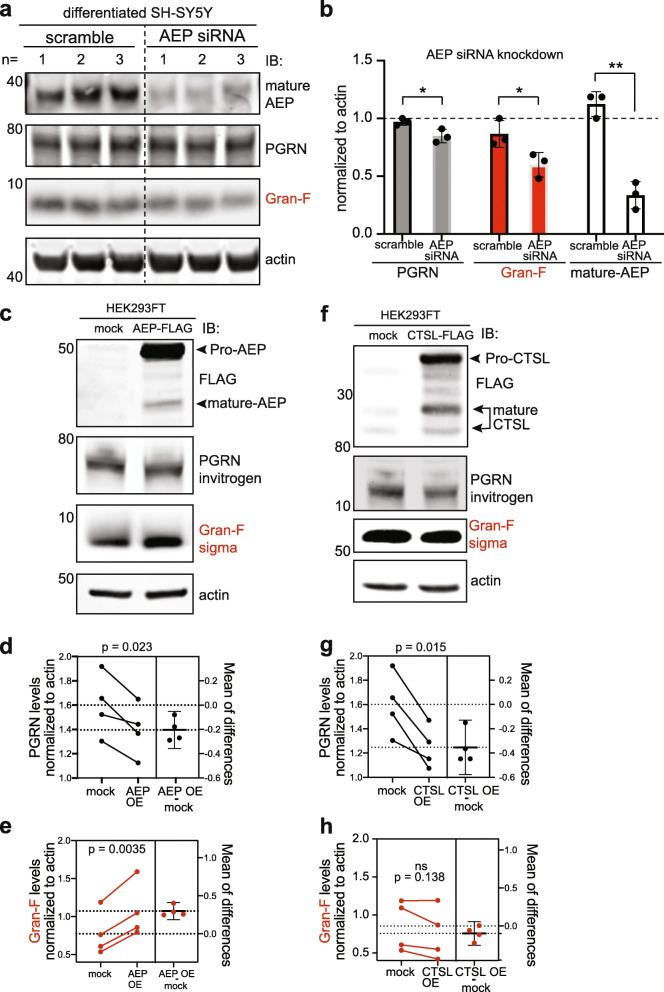
Asparagine endopeptidase (AEP) is a PGRN protease that liberates granulin F. **a-b,** Differentiated SH-SY5Y cells were treated with siRNA against AEP or scramble control for 72 h. Cells were lysed and western blotting performed for endogenous AEP, PGRN or Gran-F as indicated. Mature AEP, PGRN and Gran-F were significantly decreased in AEP siRNA treated cells (*p < 0.04, **p = 0.0010) relative to actin. Unpaired student’s t-test, error bars represent mean with standard deviation, *n* = 3. **c,** HEK293FT cells were transiently transfected with FLAG-tagged AEP (AEP FLAG or AEP OE) or FLAG alone (mock) for 24 h and cells lysates were probed for endogenous PGRN and Gran-F. AEP expression was confirmed using an anti-FLAG antibody. Each biological replicate was run on a separate western blot and normalized to actin. **d-e,** Quantification of **c** showing that overexpression of AEP decreased endogenous PGRN and increased Gran-F levels. Paired t-test analysis, *p* values are indicated, error bars represent mean with standard deviation, *n* = 4. **f,** HEK293FT cells were transiently transfected with FLAG-tagged CTSL (CTSL FLAG) or FLAG alone (mock) for 24 h and cells lysates were probed for endogenous PGRN and Gran-F. CTSL expression was confirmed using an anti-FLAG antibody. Each biological replicate was run on a separate western blot and normalized to actin. **g-h,** Quantification of **f** showing that overexpression of CTSL decreased endogenous PGRN but did not alter Gran-F levels. Paired t-test analysis, p values are indicated, error bars represent mean with standard deviation, n = 4

To study the processing of PGRN in vivo in cells, we chose SH-SY5Y cells for their ability to be terminally differentiated into neuron-like cells. These cells produce detectable amounts of both endogenous PGRN and granulin F (Fig. 3a). We first determined the expression profile of PGRN-cleaving proteases by qPCR analysis. We found that the lysosomal proteases CTSB, CTSL, CTSK, CTSS, AEP and CTSE were expressed in differentiated SH-SY5Y cells, but CTSV and CTSG were not detected (Fig. S8a).

Since AEP is the only protease that robustly liberated granulin F in vitro, and granulin F is the only granulin that can be reliably detected from cell lysates [19], we next sought to further validate AEP as a PGRN protease in these cells. To determine if AEP cleaves PGRN endogenously in terminally differentiated SH-SY5Y cells, we performed siRNA knock down of AEP and assessed the levels of endogenous PGRN and granulin F in terminally differentiated SH-SY5Y cells. Consistent with our in vitro results, knock down of AEP resulted in a significant decrease in the levels of granulin F compared to scramble-treated controls (Fig. 3a and b). While full-length PGRN was also modestly decreased, the degree to which it declined was less than that of granulin F. In addition, when tested with another anti-progranulin antibody, PGRN levels did not significantly change (Fig. S8b-d).

To further assess AEP cleavage of PGRN, we transiently over-expressed FLAG-tagged AEP or CTSL in HEK293FT cells, which have improved transfection efficiencies. Over-expression of AEP led to a significant decrease in the level of full-length PGRN and a corresponding increase in the granulin F compared to control (Fig. 3c-e). In contrast, over-expression of CTSL, which digests PGRN in vitro but was not shown to release Gran F, led to a significant decrease in full-length PGRN without an increase granulin F (Fig. 3f-h). This suggests that in cells, both AEP and CTSL can cleave full length PGRN but only AEP liberates granulin F.

**Fig. 4:**
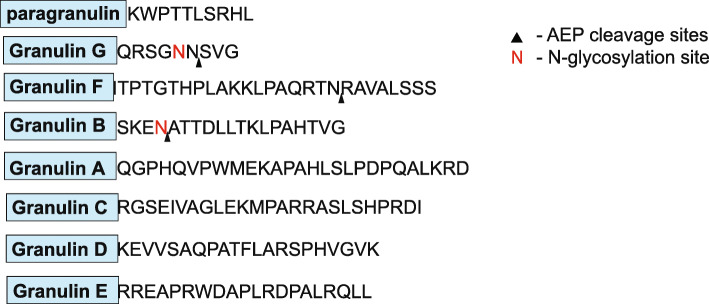
Asparagine endopeptidase liberates granulin F from PGRN. Visual representation of progranulin protein with individual granulin domains annotated in blue boxes with their associated C-terminal inter-granulin linker following. AEP cleavage sites are indicated with black arrowhead . N, represents N-linked glycosylation sites

To determine the specific cleavage sites of AEP, we incubated recombinant human PGRN with and without AEP for 60 min at pH 4.5. Bands corresponding to full-length PGRN, multi-granulin bands, and granulin-sized bands were observed by silver staining (Fig. S9a). These bands were subjected to LC-MS/MS to determine AEP cleavage sites. Consistent with its activity as a protease that cleaves after asparagine residues, we identified three sites for AEP cleavage within three inter-granulin linkers (G-F, F-B, B-A) that would result in the liberation of granulins F and B from PGRN (Fig. 4, Fig. S9b-e). Furthermore, a previous study identified CTSL cleavage sites on PGRN and showed that CTSL cannot liberate granulins F and B due to lack of cleave sites in those inter-granulin domains [17].

### Granulins F levels are increased in the degenerating regions of the brains from FTLD-TDP-*Pgrn* subjects

PGRN haploinsufficiency predisposes to FTLD-TDP. Interestingly, in *C. elegans*, the processing of progranulin to granulins increases with age [46]. Further, the presence of an MGF containing granulin E is decreased in FTLD-TDP [12]. However, whether PGRN processing into granulin F is affected in FTLD, an age-related disorder, is unclear. To assess whether PGRN processing is altered in FTLD, we measured granulin F levels from brain samples of control individuals or those with FTLD due to *Pgrn* mutations (FTLD-TDP-*Pgrn)*. As previously described [12], we examined a brain region affected in FTLD (middle frontal gyrus, MFG, defined as gliosis score of 3) as well as an unaffected region (inferior occipital cortex, IOC, defined as gliosis score of 0–1) from both groups. Characteristics of subjects are shown in Table 2.

**Table 2:**
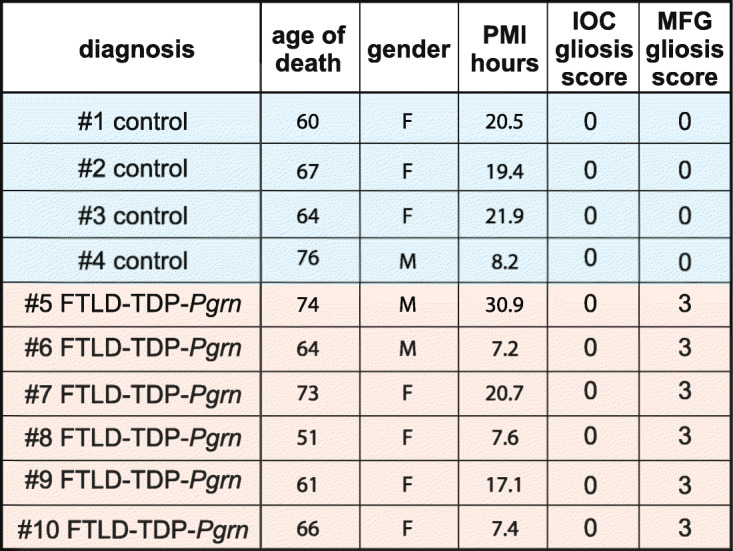
Clinical Information for control and FTLD-TDP-*Pgrn* subjects.

**Fig. 5:**
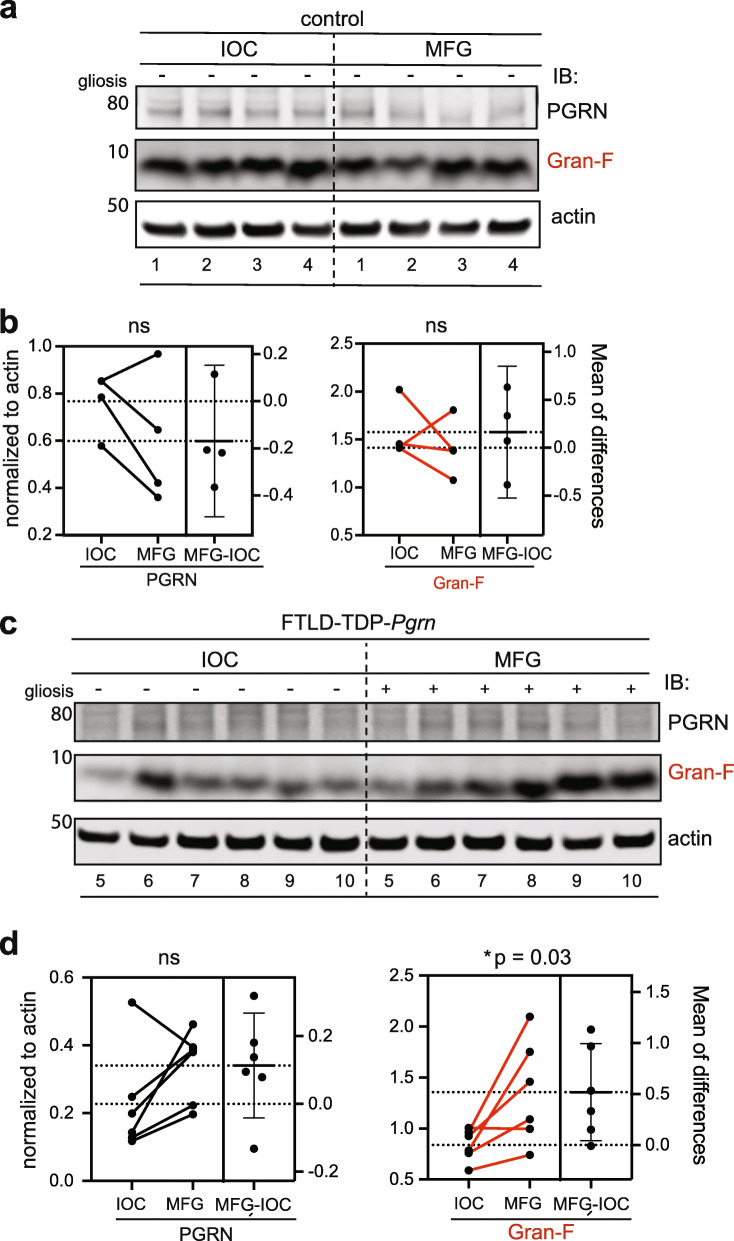
Granulins F levels in FTLD-TDP-*Pgrn* subjects versus controls. **a**, Western blot analysis of endogenous PGRN and Gran-F in IOC and MFG regions of control human brain. **b**, Quantification of PGRN and Gran-F levels in IOC and MFG regions in control subjects (ns, not significant). **c**, Western blot analysis of endogenous PGRN and Gran-F in IOC and MFG regions of FTLD-TDP-*Pgrn* subjects. **d**, Quantification of PGRN and Gran-F levels in IOC and MFG regions in FTLD-TDP-*Pgrn* subjects (*, *p* = 0.03). Error bars represent mean with standard deviation. Paired student-t test was performed between each pair. Sample numbers listed below each Western blot correspond to subject number in Table 2

Clinical information for the control and FTLD-TDP-*Pgrn* subjects. PMI, post-mortem interval; IOC, inferior occipital cortex; MFG, middle frontal gyrus. Sample numbers correspond to Figs. 5 and S10

First, we compared the levels of PGRN and granulin F between the degenerating MFG and the non-degenerating IOC regions of the control and FTLD-TDP-*Pgrn* brain. No significant difference was noted in the levels of PGRN and Gran-F between IOC and MFG of control subjects (Fig. 5a-b). However, Gran-F levels were significantly elevated in MFG vs IOC of FTLD-TDP-*Pgrn* subjects (Fig. 5c-d). We also compared the levels of PGRN and granulin F between control and FTLD-TDP*-Pgrn* subjects in the same regions. As expected, full-length PGRN levels are lower in FTLD-Pgrn cases versus controls, but in this comparison, granulin F is not significantly different (Fig. S10).

**Fig. 6:**
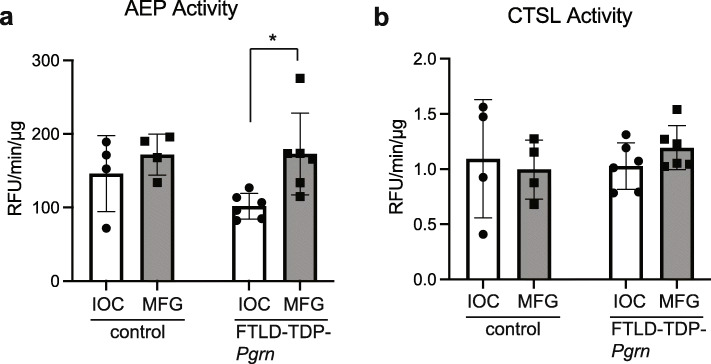
AEP activity in FTLD-TDP-*Pgrn* subjects versus controls. AEP (**a**) and CTSL (**b**) activity are compared between the degenerating middle frontal gyrus (MFG) and the non-degenerating inferior occipital cortex (IOC) regions in control versus FTLD-TDP-*Pgrn* subject neuropathological samples (*, *p* = 0.013). Unpaired two-tailed student t-test was used to compare. Error bars represent mean with standard deviation

Since AEP was the only enzyme that could robustly liberate granulin F in our in vitro assays and affected the levels of granulin F in cells (Figs. 2 and 3), we quantified the activity of AEP in both regions of the brain. These assays revealed a significant increase in AEP activity in disease affected MFG compared to non-affected IOC from the same subjects (Fig. 6a). Furthermore, we see a similar trend towards an increased level of mature AEP in the degenerating MFG region compared to the non-affected IOC regions of FTLD-TDP-*Pgrn* subjects but not controls (Fig. S11). To determine if the change in levels and activity AEP was also seen for other known PGRN proteases, we assayed the activity of CTSL. Interestingly, there was no significant change to CTSL activity in either regions across groups, suggesting differential effects on lysosomal enzymes in FTLD (Fig. 6b).

These data suggest that increased AEP activity in the degenerating MFG region may alter PGRN processing in a way that leads to an increase in production of granulin F in that region. We were unable to detect other granulin peptides but had previously shown in both FTLD-TDP subjects negative for *Pgrn* mutations and Alzheimer’s disease brain that a multi-granulin fragment containing granulin E was also increased in a diseased brain region [12]. Thus, it may be that these neurodegenerative diseases lead to overall increased processing of PGRN into bioactive granulins.

## Discussion

This study sought to better understand all aspects of PGRN cleavage into granulins, from the proteases involved and pH-dependence of cleavage to the MGFs/granulins produced and relevance to human disease. The normal functions of PGRN as an activator of CTSD are slowly being elucidated [14]. However, granulins are less well-understood. Since full-length, partially cleaved and granulins are all bioactive [14], a complete and nuanced understanding of their production and levels will likely be critical to understanding normal, balanced PGRN function and how this change with age and disease. We have shown that in addition to CTSL and CTSB, multiple proteases can process PGRN and liberate granulins, and all of these proteases act in a pH-dependent manner. Additionally, these proteases have differential expressions in cells and tissues. CTSB, CTSL and AEP are expressed in all cells and tissues including the brain, however CTSS, CTSK, CTSV, CTSE and CTSG show cell- and tissue-specific expression [2, 32, 44, 45]. This suggests that PGRN processing may be regulated in a cell-type specific manner and future studies in cells that endogenously express these proteases may help better understand their roles.

Although our in vitro assays identified individual proteases capable of cleaving PGRN into granulins, we recognize several limitations of these types of assays. First, they lack key PGRN binding partners, such as prosaposin and potential co-factors that can modulate protease activity, such as peptide inhibitors like cystatins and aspartins. Secondly, PGRN in lysosomes will likely be simultaneously cleaved by multiple proteases that also interact with one another [47], which is not simulated in this approach. However, to gain clarity as to the complement of lysosomal proteases that are able to cleave PGRN, this study is the first and most comprehensive of its kind. The ability to differentiate between MGFs of various molecular weights, pH optima, and relative protease cleavage efficiency are other advantages to our approach.

While we and others have shown alterations in granulin levels in FTLD-TDP [12, 19], this study is the first study to implicate a change in a specific protease, AEP. To our surprise, these results also demonstrated relatively higher granulin F levels in an area of severe neurodegeneration in human FTLD-FTD-*Pgrn* cases compared to a region with little to no degeneration. Previously, Holler et al., 2017, demonstrated a reduction in certain granulins in brain, however, the severity of degeneration and astrogliosis within these regions was not assessed [19]. Since degenerating regions are characterized by neuronal loss and infiltration of inflammatory astrocytes and microglia, the latter of which expresses high levels of AEP [45], it is possible that glial cells, such as astrocytes or microglia, are responsible for the observed increase in granulin F. Moreover, since PGRN is also a secreted protein, we cannot yet differentiate the cell autonomous and cell non-autonomous contributions of PGRN processing in disease. Whether the increased granulin F contributes to or results from FTLD pathophysiology also remains to be seen. AEP has previously been reported to cleave aggregate-forming proteins, such as amyloid precursor protein (APP), microtubule associated protein tau (MAPT) and TAR-DNA binding protein 43 (TDP43) and is also implicated in an FTLD-related disorder, amyotrophic lateral sclerosis (ALS) [32, 48–51]. Interestingly, AEP is dysregulated and activated during aging, and AEP activity is increased in regions of degeneration in AD human brain and mouse models suggesting that PGRN processing and granulin F production may be altered in other neurodegenerative diseases as well [49]. If future studies implicate a causal role for granulin F in FTLD and other diseases, targeting AEP to decrease the levels of granulin F in the degenerating regions of the brain may be neuroprotective.

In summary, our study suggests that PGRN processing into granulins undergoes multiple levels of regulation, including protease-specific cleavage sites in the various inter-granulin linkers and differential cleavage based on pH setpoints, which may reflect activity in different endo-lysosomal compartments. Still to be explored are cell-type and temporal regulation of PGRN cleavage. Given the highly evolutionarily conserved presence of PGRN and granulins, as well as the gene mutations associated with disease, intricate regulation indicates that PGRN and granulin levels are important for maintaining cellular homeostasis and function. Our studies have helped identify multiple players that contribute to this homeostasis, providing new avenues to regulate the levels of granulins in disease and placing the broad and contrasting roles of granulins into perspective.

## Conclusion

This study comprehensively identifies a suite of endo-lysosomal proteases that can process full-length PGRN into multi-granulin and single granulin fragments. These proteases have strikingly specific cleavage patterns and optimal pH settings. The complexity of PGRN cleavage could confer multiple layers of cell-type and environmentally responsive regulation upon PGRN processing that can be altered with age and disease. As full-length, multi-granulin and single granulin fragments likely have different biological activities, these findings are highly relevant in understanding the consequences of progranulin replacement therapies and could be leveraged to promote neuronal health through inhibition of specific PGRN proteases.

## Supplementary Information

**Additional file 1 **Fig. S1. Enzymes that do not cleave PGRN are biologically active. a, 1 μM of mature CTSD was incubated with 400 ng recombinant human PGRN or 400 ng BSA at the indicated pH. The incubation was stopped after 60 min or 16 h, and reaction contents were subjected to Coomassie staining (60 min) or silver staining (16 h) respectively. Bands correlate to proteins indicated on the right. BSA was used as a control for CTSD activity. At lower pH of 3.4, CTSD self-digest leading to a decreased level of mature CTSD compared to pH 4.5 [52]. b, FRET-based enzyme activity assay at pH 4.5 was performed on all enzymes that do not cleave PGRN. The values obtained were normalized to timepoint t = 0 and plotted relative to activity. All enzymes show activity against the optimized FRET peptides compared to substrate only control. Fig. S2. Antibody validation for individual granulin peptides. a, Dot blots confirming the specificity of each custom antibody towards the granulin peptide. 2.5 μg of each peptide was used with each antibody (11,000 dilution). Paragranulin, p-Gran; granulin G, Gran-G; granulin F, Gran-F; granulin B, Gran-B; granulin A, Gran-A; granulin C, Gran-C; granulin D, Gran-D; granulin E, Gran-E. b, Peptide blocking experiment to demonstrate antibody specificity. 400 ng of rhPGRN was incubated with 1uM CTSL for 20 min. The antibody was incubated with or without the respective blocking peptide (10 or 100-fold molar ratio of peptide to antibody) and then incubated with the membrane overnight. Increase in peptide concentrations show a decrease in the bands of each antibody tested. Fig. S3. Lysosomal proteases unable to digest PGRN in vitro*.* Full western blot images of the proteases that do not cleave PGRN. 1 μM of each enzyme was incubated with 400 ng of PGRN for 20 min. C-terminal Gran-E antibody or anti-PGRN (Invitrogen) antibody was used to assess the results of the in vitro assay. a, Cysteine proteases Cathepsins C (CTSC), H (CTSH), X (CTSX), O (CTSO) and F (CTSF). b, Aspartyl proteases cathepsin D (CTSD). c, Serine protease Pro-X carboxypeptidase (PRCP) and Cathepsin A (CTSA). The lower molecular weight bands (indicated by *) correspond to the enzyme. Fig. S4. PGRN processing by lysosomal proteases in vitro at pH 6.5. 1 μM of each enzyme was incubated with 400 ng of PGRN for 20 min at pH 6.5. Anti-p-Gran, anti-Gran-F, and anti- Gran-E antibodies were used to assess the results of the in vitro assay. Cathepsin B (CTSV), cathepsin L (CTSL), cathepsin K (CTSK), cathepsin S (CTSS), cathepsin V (CTSV), asparagine endopeptidase (AEP), cathepsin G (CTSG), paragranulin (p-Gran), granulin F (Gran-F), granulin E (Gran-E). Fig. S5. Summary of PGRN processing into granulins by multiple proteases. Summary of the results of the in vitro assays. a, Processing of PGRN to granulins by different proteases is dependent on pH. The range of cleavage is represented in greyscale with no cleavage in white and complete cleavage in black. The range takes into account the amount of full-length PGRN processed into multi-granulin fragments and individual granulins within 20 min. b, Represented is the ability of each enzyme to liberate individual paragranulin (p) in red, granulin F (F) in green, and granulin E (E) in blue. The protease classes are also indicated. CTSE, cathepsin E; CTSV, cathepsin V; CTSL, cathepsin L; CTSB, cathepsin B; CTSK, cathepsin K; AEP, asparagine endopeptidase; CTSG, cathepsin G; CTSS, cathepsin S. Fig. S6. CTSL is highly efficient at liberating paragranulin and granulin E from PGRN. A time course analysis to determine efficiency of cleavage. 400 ng of recombinant human PGRN was incubated with 50 nM of enzyme for the time points indicated. P-Gran, Gran-F and Gran-E antibodies were used to assess the results of the assay. PGRN, progranulin; CTSL, cathepsin L; CTSB, cathepsin B. Fig. S7. Antibody specificity to detect both PGRN and granulin sized bands in wild-type and PGRN knock out iPSC cell lysates. a, Lysates from isogenic WTC11 and *Pgrn* KO iPSCs were probed with custom anti-granulin antibodies. b, Commercial antibodies from Invitrogen and Sigma were tested on the same iPSC cell lines. Fig. S8. Expression profile of the PGRN proteases in differentiated SH-SY5Y cells. a, qPCR analysis to assess the expression of candidate PGRN proteases in differentiated SH-SY5Y cells. The graph represents the mean expression value of each enzyme normalized to GAPDH. The absolute mean values are noted for each sample. All enzymes were run in triplicates with *n* = 4 biological replicates. b, Differentiated SH-SY5Y cells were subjected to treatment with AEP siRNA or scramble control. A separate quantification and statistical analysis of PGRN, Gran-F and mature AEP was performed, similar to that seen in Fig. 3 of the manuscript. c, PGRN protein is assessed with an Anti-Gran E antibody. PGRN KO SH-SY5Y cells are used to determine specificity of PGRN band. d, Quantification of c. Fig. S9. PGRN is processed by AEP to liberate individual granulins F and B. Recombinant human PGRN was incubated with and without AEP for 1 h at pH 4.5. The reaction was stopped, and the cleavage bands were separated by SDS-PAGE. a, Multiple cleavage bands and individual granulin sized bands can be visualized upon silver stain. b-e, Bands were cut and digested for mass spectrometry to identify the cleavage sites (also see Fig. 4c). Identified peptides are annotated with observed fragment ions. Previous and next amino acids are in () and carbamidomethylated Cys are colored red. The precursor mass of each peptide is as follows: b, 428.5229 m/z; c, 847.8607 m/z; d, 639.3205 m/z; e, 478.9133 m/z. Fig. S10. Progranulin and granulin F levels in the human brain. a, Western blot analysis of the levels of PGRN and Gran-F in the non-degenerating IOC regions of the control brain compared to the same region in FTLD-TDP-*Pgrn* subjects. b, quantification of PGRN and Gran-F level, normalized to actin. c, Western blot analysis of the levels of PGRN and Gran-F in the degenerating MFG regions of the control brain compared to the same region in FTLD-TDP-*Pgrn* subjects. d, quantification of PGRN and Gran-F level, normalized to actin. (**, *p* = 0.008). Unpaired, two-tailed student t-test was performed between the pairs. Error bars represent mean with standard deviation. e-g, Levels of PGRN and Gran-F in a and c are plotted against age. There is no correlation between age and level of PGRN and Gran-F. Fig. S11. AEP levels are significantly increased in a degenerating brain region of FTLD-TDP-*Pgrn* subjects. a, Western blot analysis of endogenous immature (pro AEP) and mature AEP levels in control brain. b, Quantification of pro and mature AEP levels between IOC and MFG regions in control subjects. Paired two-tailed student t-test was performed between the different groups. No significant (ns) difference was observed. c, Western blots of endogenous Pro and mature AEP in IOC and MFG brain regions of FTLD-TDP-*Pgrn* subjects. d, Levels of mature AEP in the degenerating MFG region are increased compared to the non-degenerating IOC region from the same FTLD-TDP-*Pgrn* samples (*, *p* = 0.01, *n* = 6). Paired two-tailed student t-test was performed between the groups. IOC, inferior occipital cortex; MFG, middle frontal gyrus; AEP, asparagine endopeptidase. Sample numbers listed below each Western blot correspond to subject number in Table 2.

## Data Availability

All data generated or analyzed during this study are included in this published article and its supplementary information files.

## Abbreviations

PGRN: Progranulin
Gran-F: granulin F
p-Gran: paragranulin
Gran-E: granulin E
CTSB: Cathepsin B
CTSL: Cathepsin L
CTSK: Cathepsin K
CTSS: Cathepsin S
CTSV: Cathepsin V
AEP: Asparagine endopeptidase
CTSH: Cathepsin H
CTSX: Cathepsin X
CTSC: Cathepsin C
CTSD: Cathepsin D
CTSE: Cathepsin E
CTSF: Cathepsin F
CTSO: Cathepsin O
CTSG: Cathepsin G
PRCP: Pro-X carboxypeptidase
CTSA: Cathepsin A
FTLD-TDP-*Pgrn*: Frontotemporal lobar degeneration leading to TDP-43 positive inclusions caused by progranulin haploinsufficiency
AD: Alzheimer’s disease
MAPT: microtubule associated protein Tau
IOC: Inferior occipital cortex
MFG: Middle frontal gyrus
MGF: multi-granulin fragments
