# Supplementary material for: Processing of progranulin into granulins involves multiple lysosomal proteases and is affected in frontotemporal lobar degeneration
Source: Mol Neurodegener. 2021 Aug 3;16:51. doi: 10.1186/s13024-021-00472-1 (PMC8330050; doi:10.1186/s13024-021-00472-1)

**Figure S1 - Enzymes that do not cleave PGRN are active**

**a**

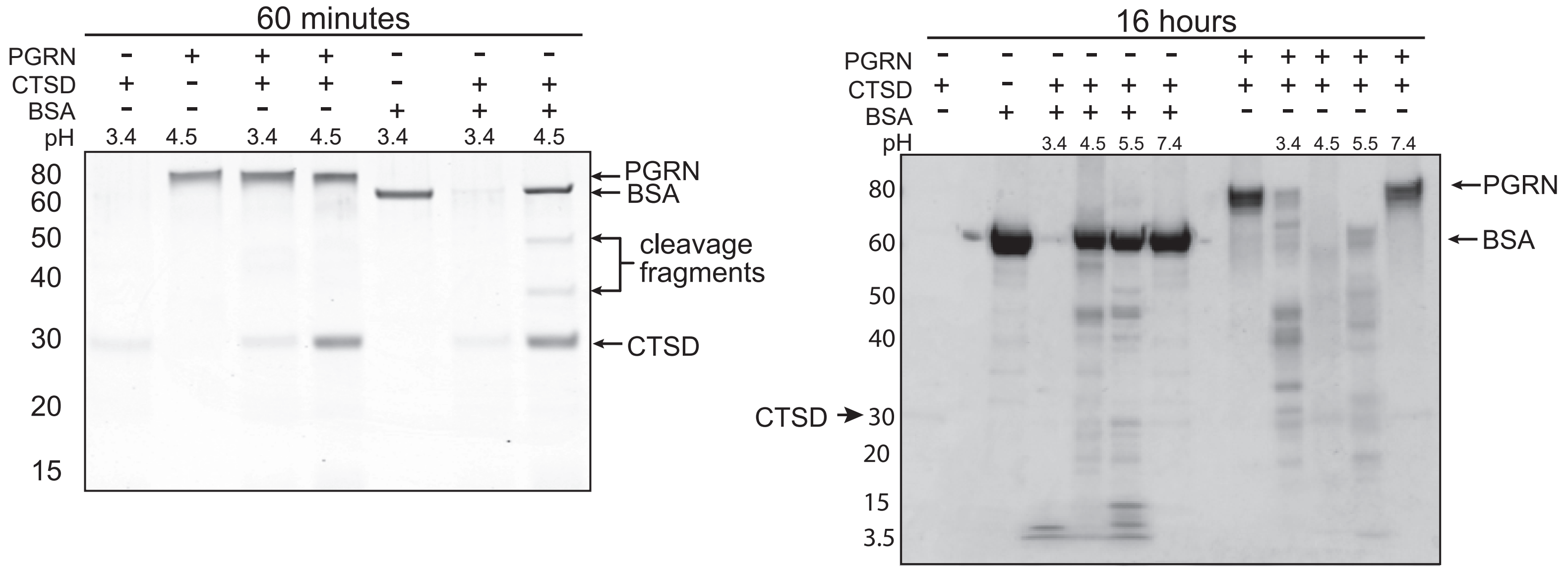


**b**

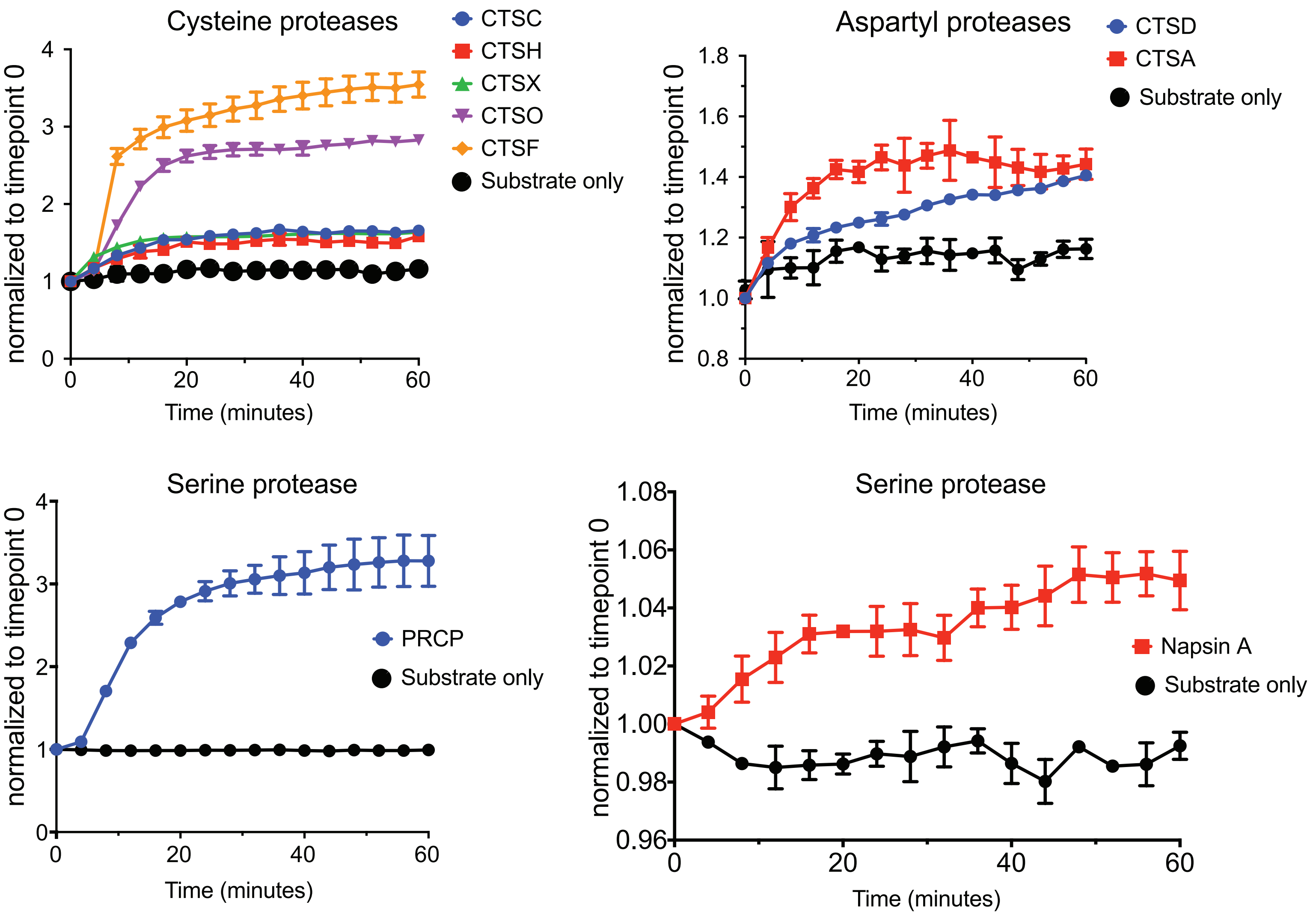

**Figure S2 - Antibody specificty to PGRN and individual granulins**

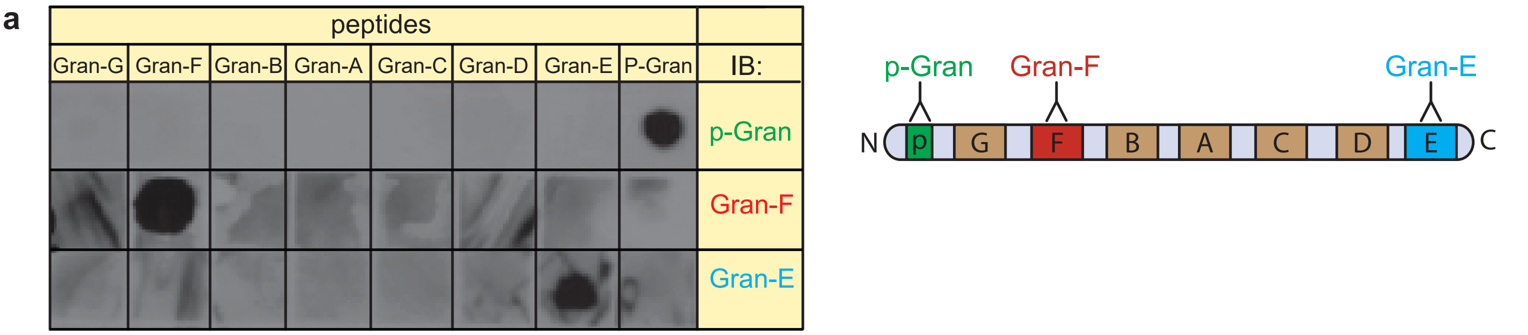


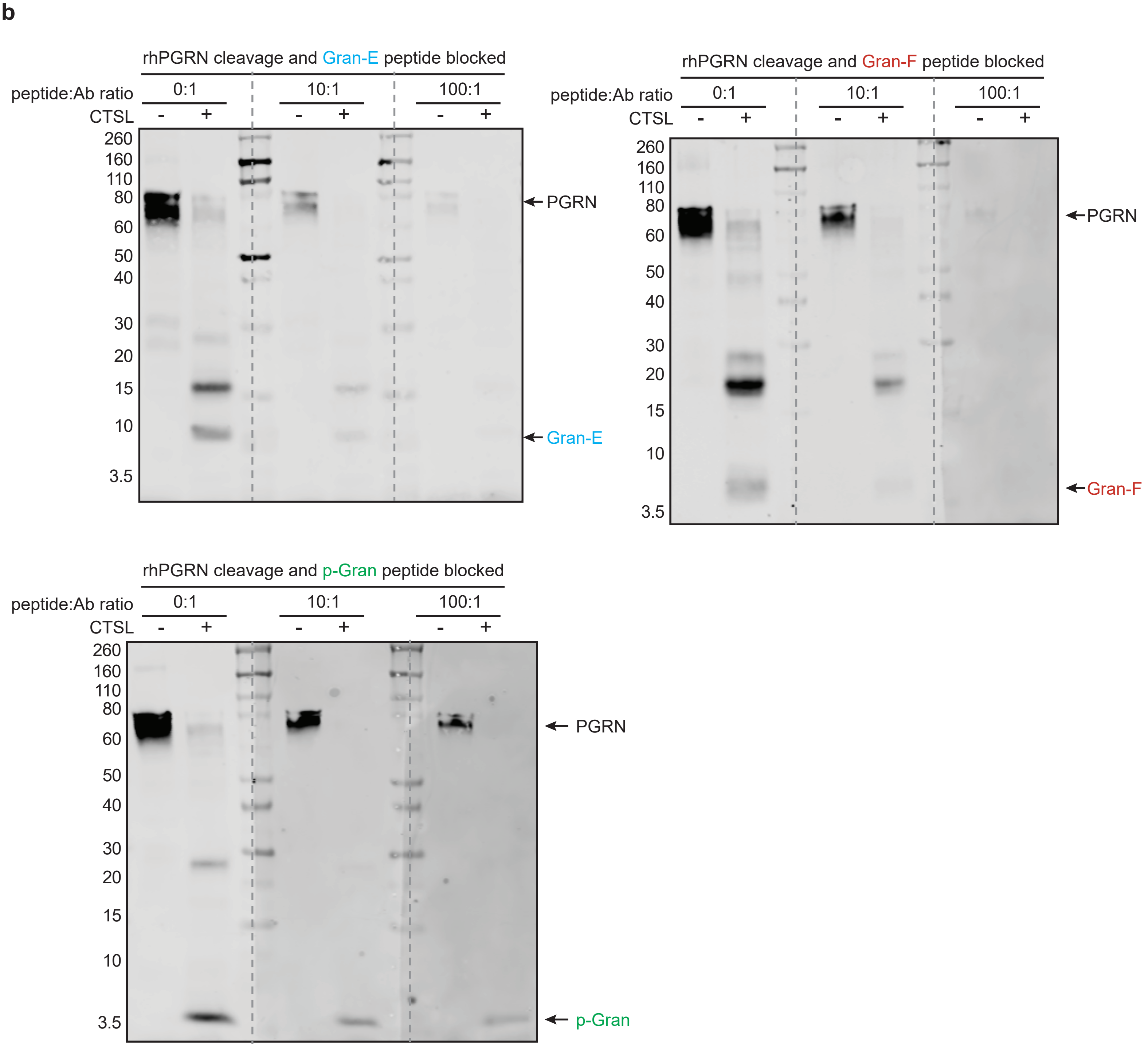

**Figure S3 - Lysosomal proteases unable to disgest PGRN *in vitro***

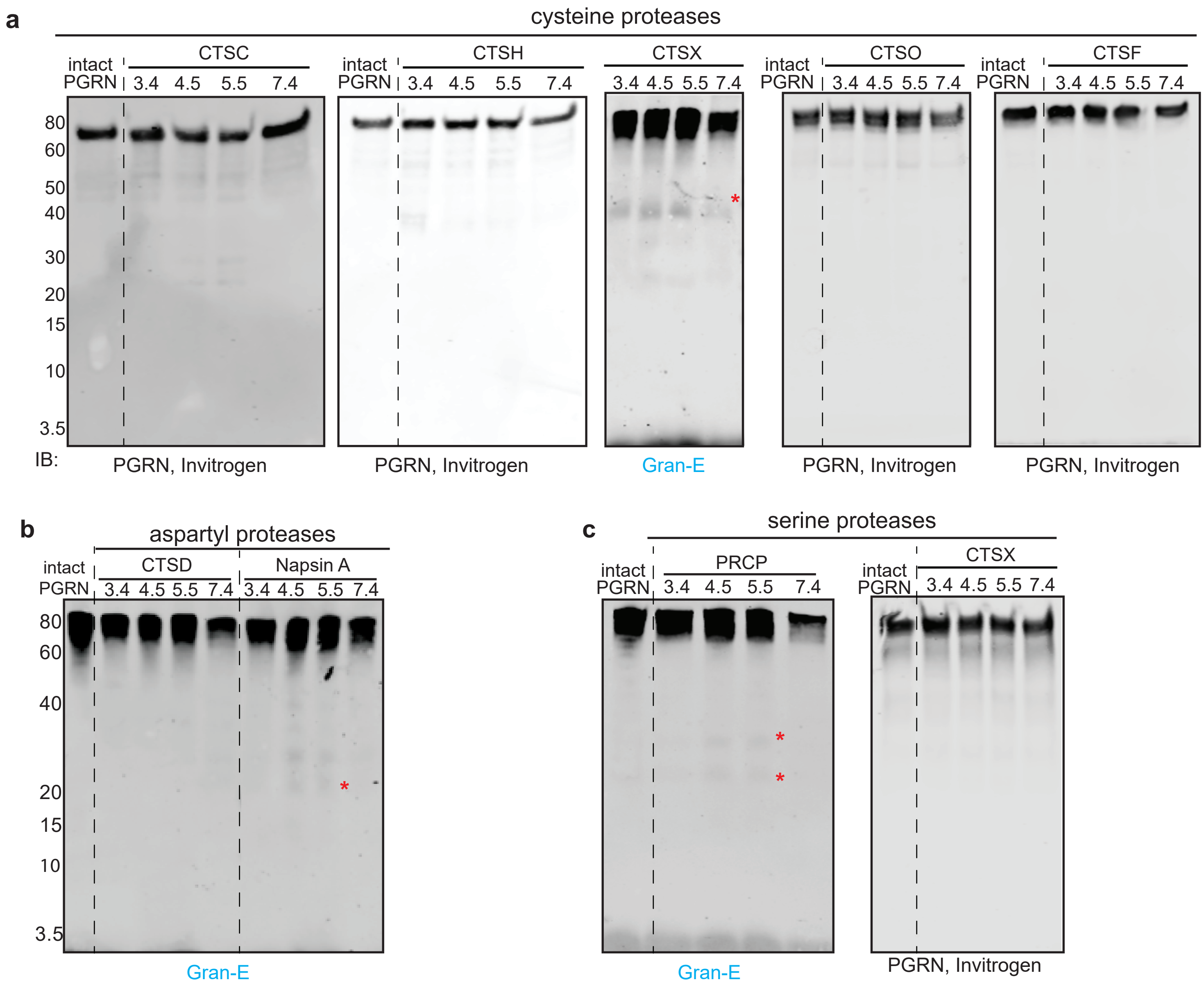

**Figure S4 - PGRN processing by lysosomal proteases *in vitro* at pH 6.5**

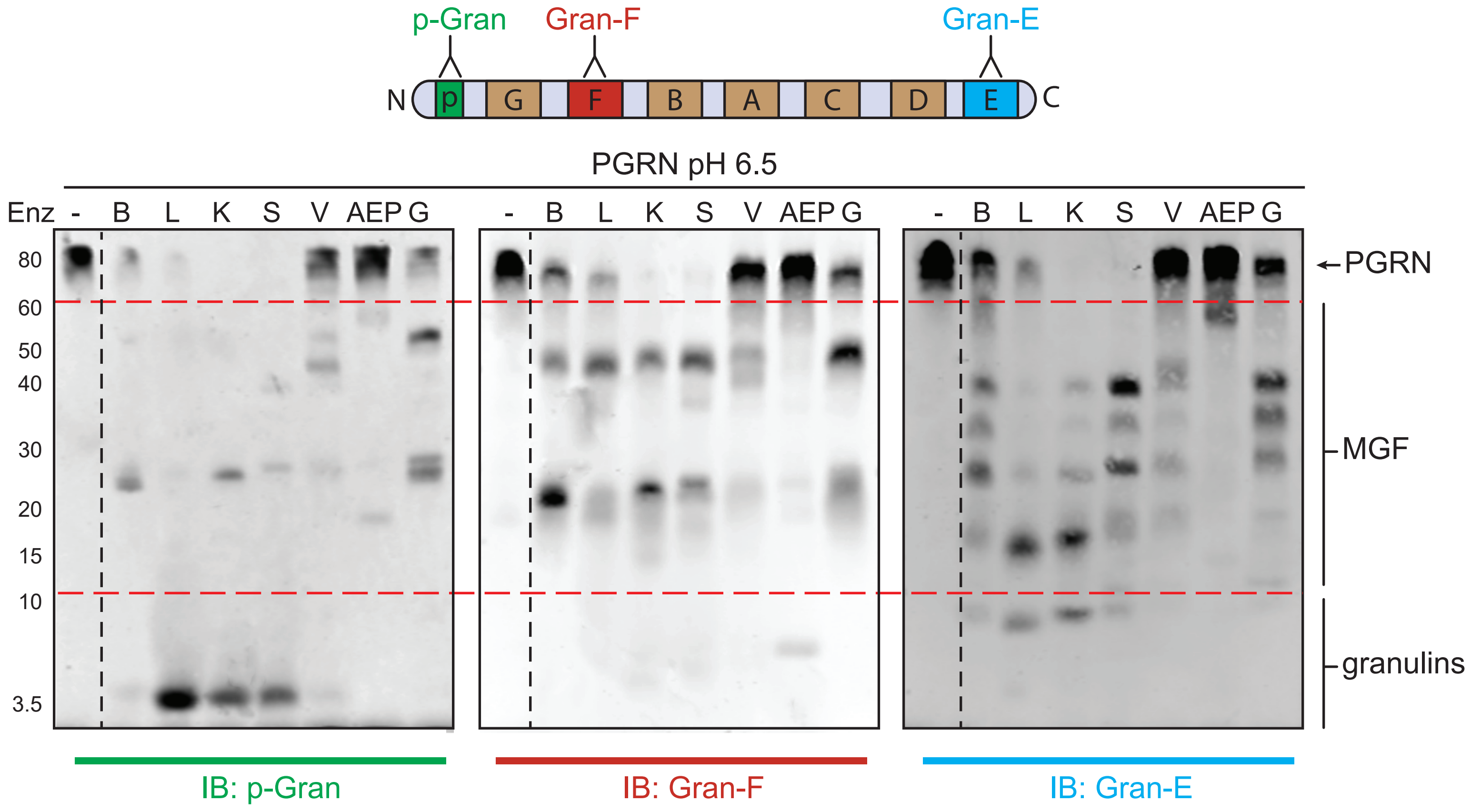

**Figure S5 - Illustrative summary of PGRN processing into granulins by multiple proteases**

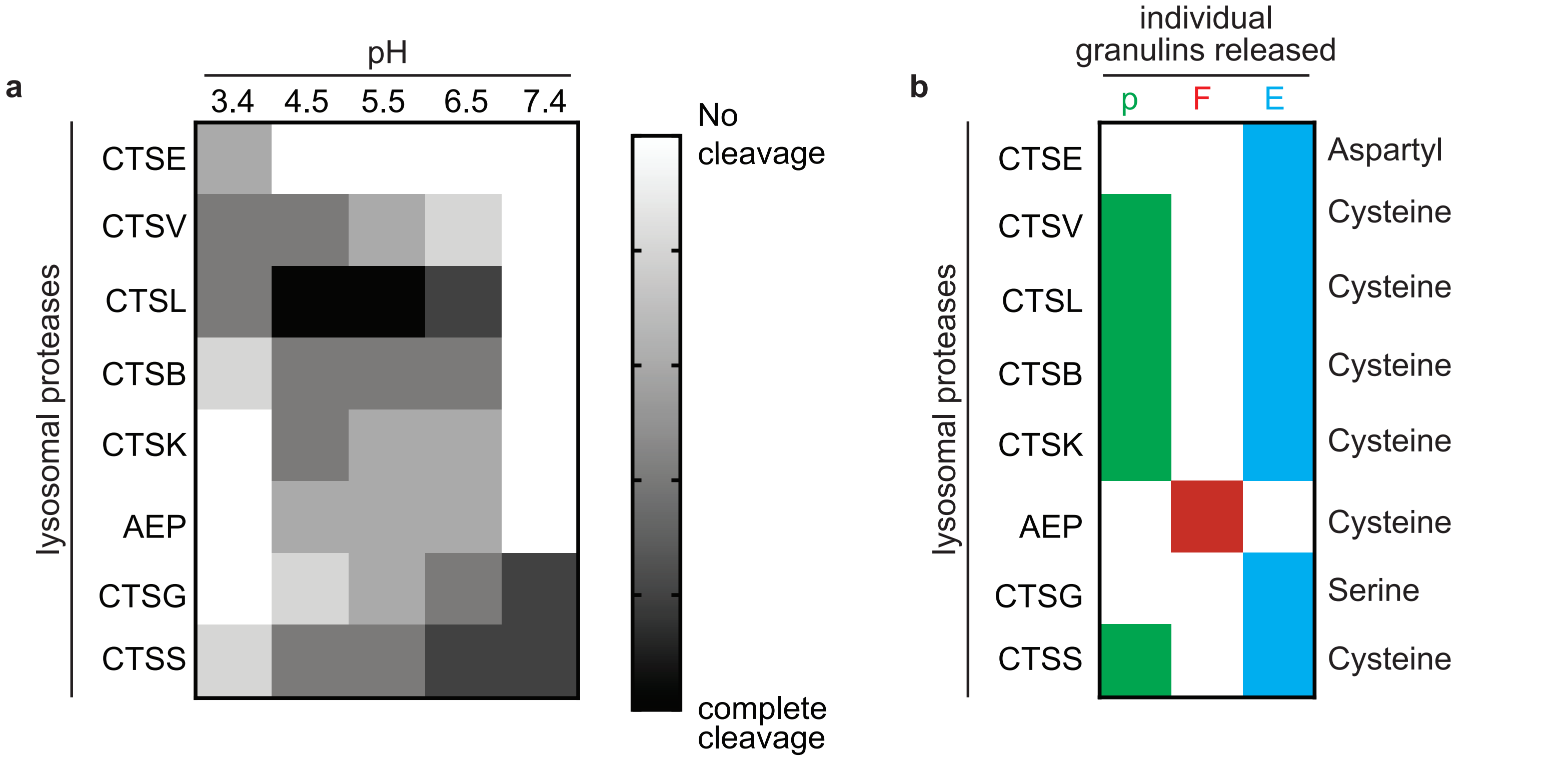

**Figure S6 - CTSL is highly efficient at liberating paragranulin and Gran-E from PGRN**

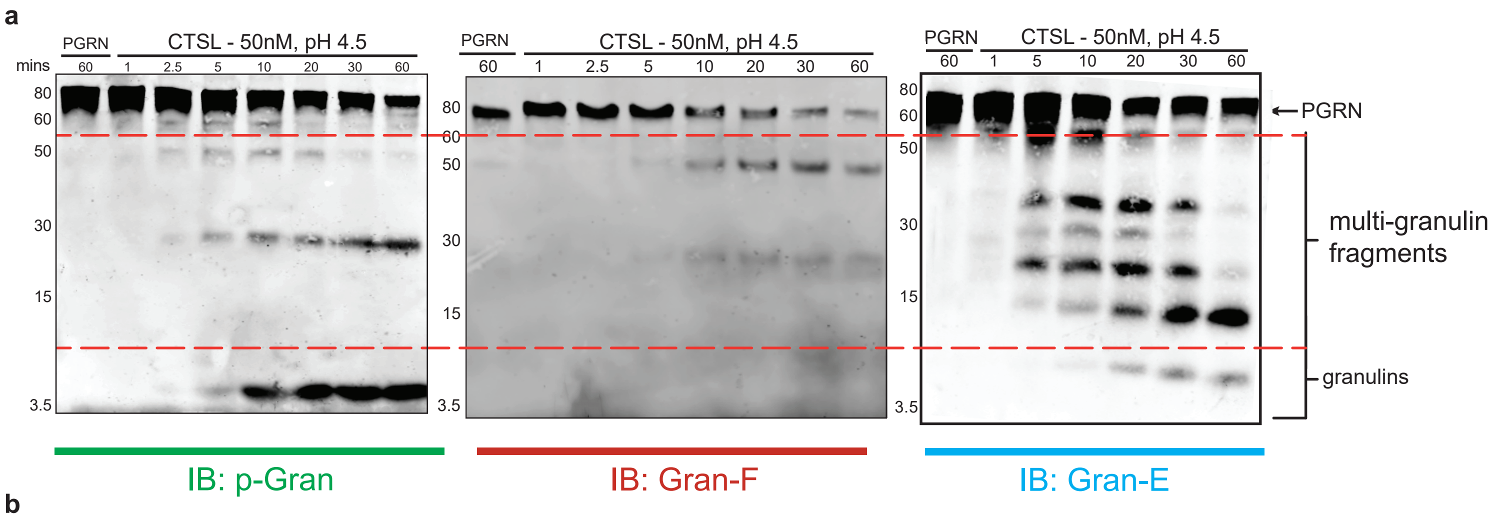


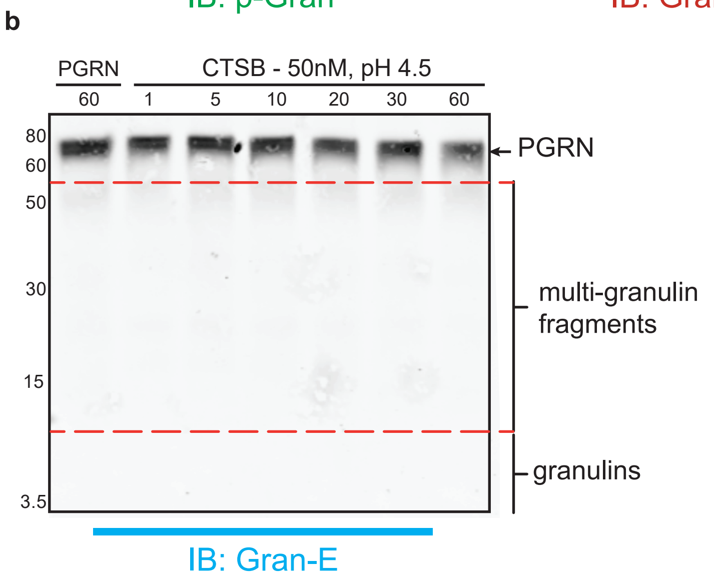

**Figure S7- Antibody specificity to detect both PGRN and granulin sized bands in iPSC lysates**

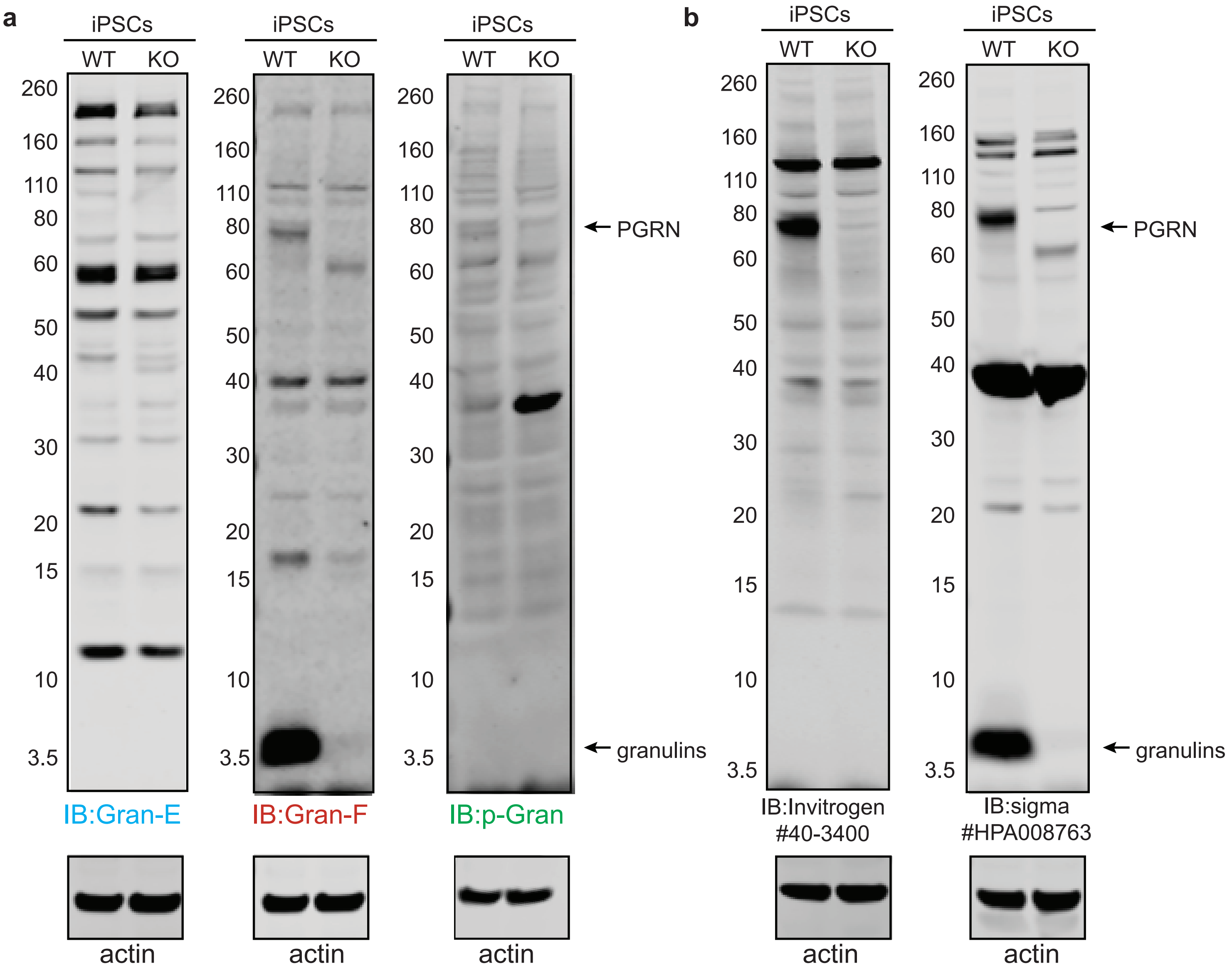

**Figure S8 - Expression profile of lysosomal proteases in differentiated SH-SY5Y cells**

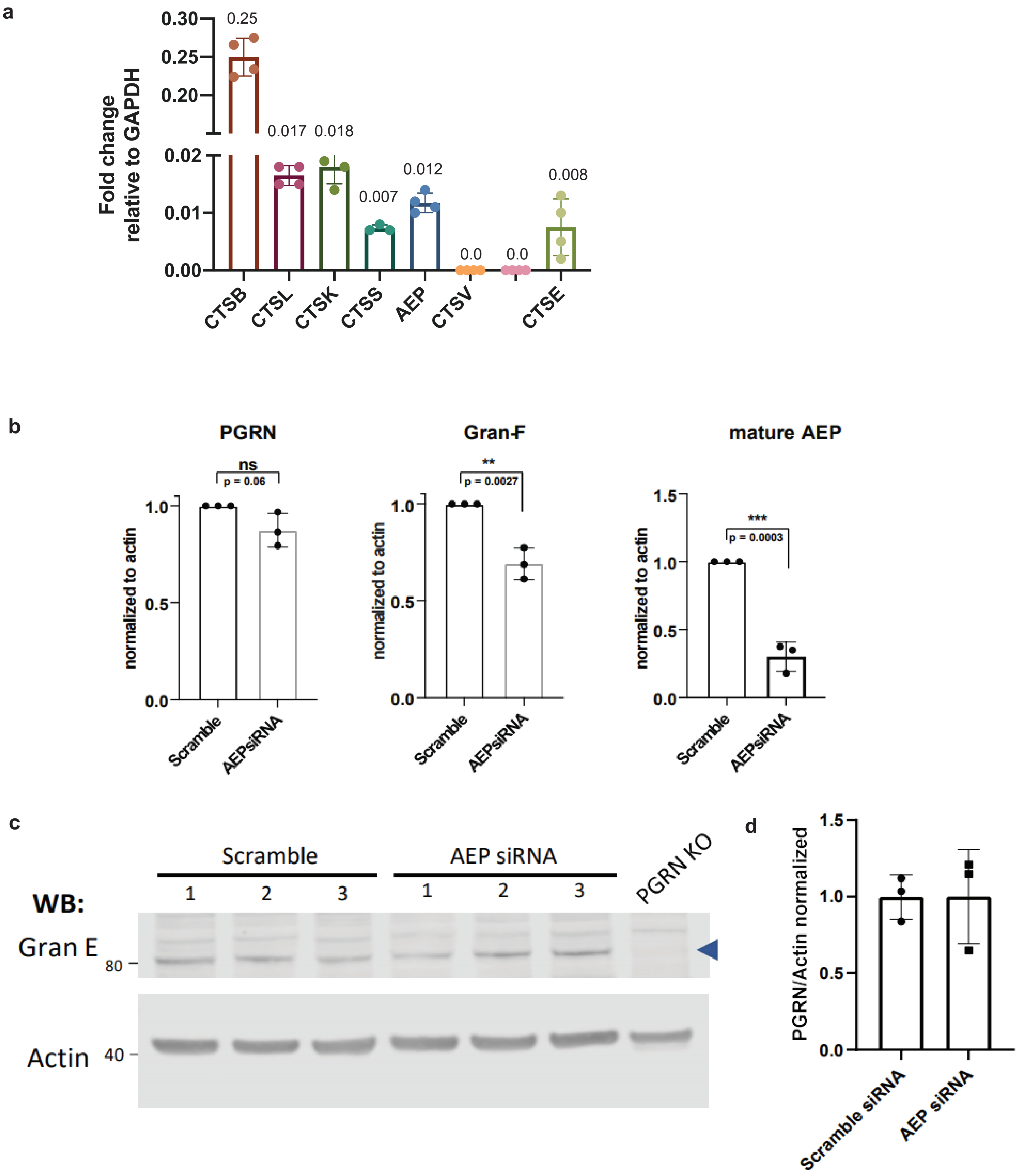

**Figure S9 - PGRN is processed by AEP to liberate individual granulins F and B**

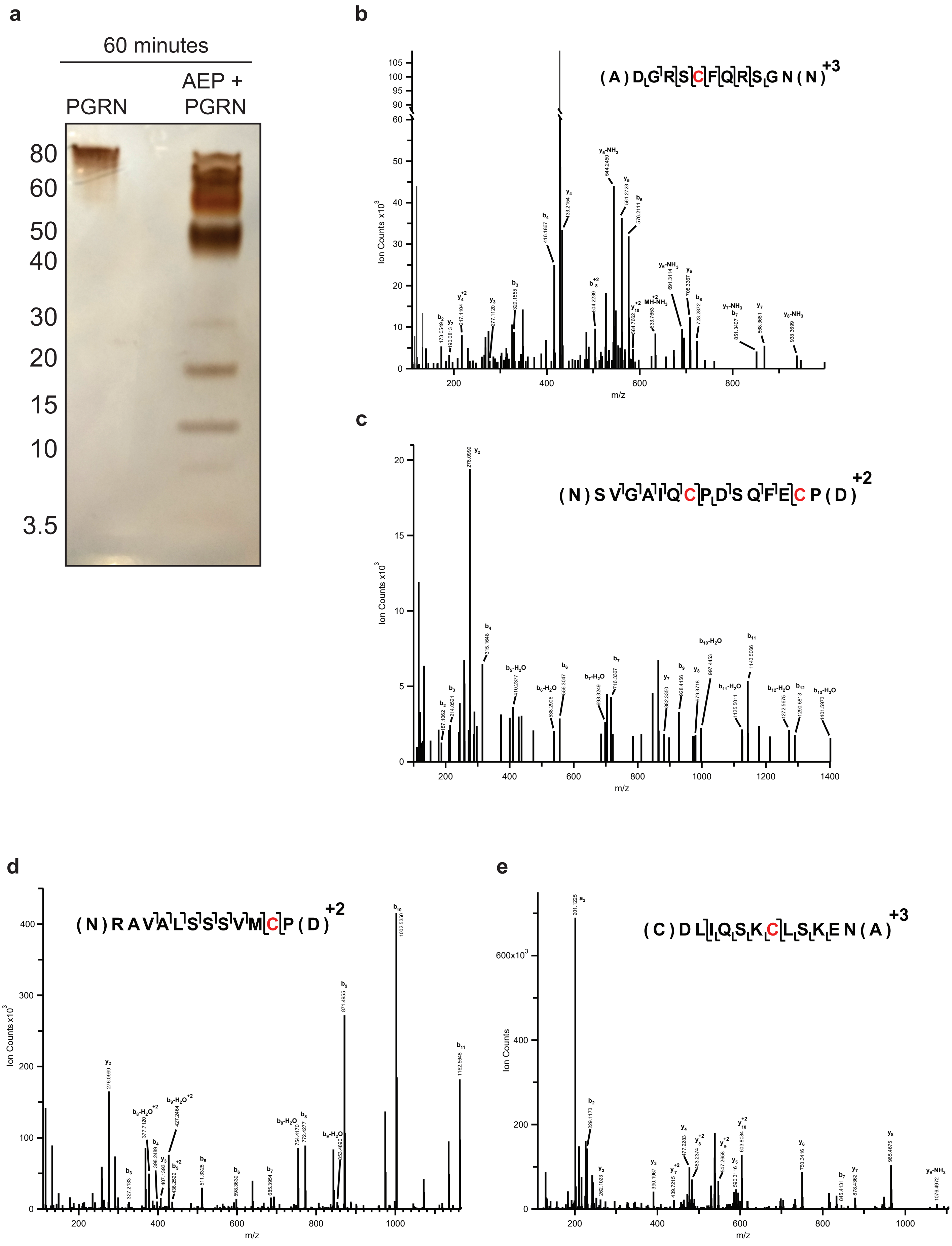

**Figure S10 - Progranulin and granulin F levels in human brain**

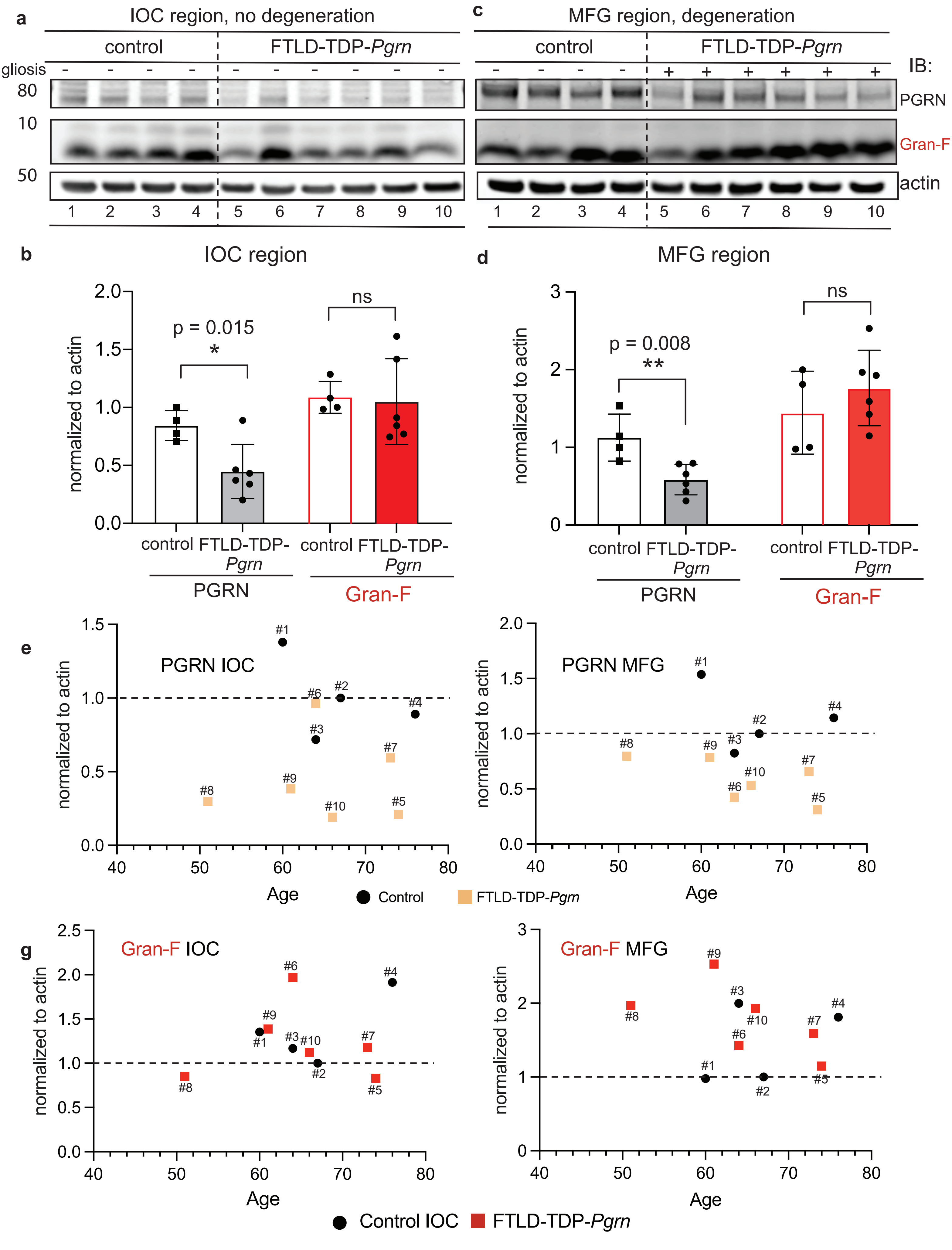

**Figure S11 - Mature AEP levels are significantly increased in degenerating regions of FTLD-TDP-*Pgrn* patients**

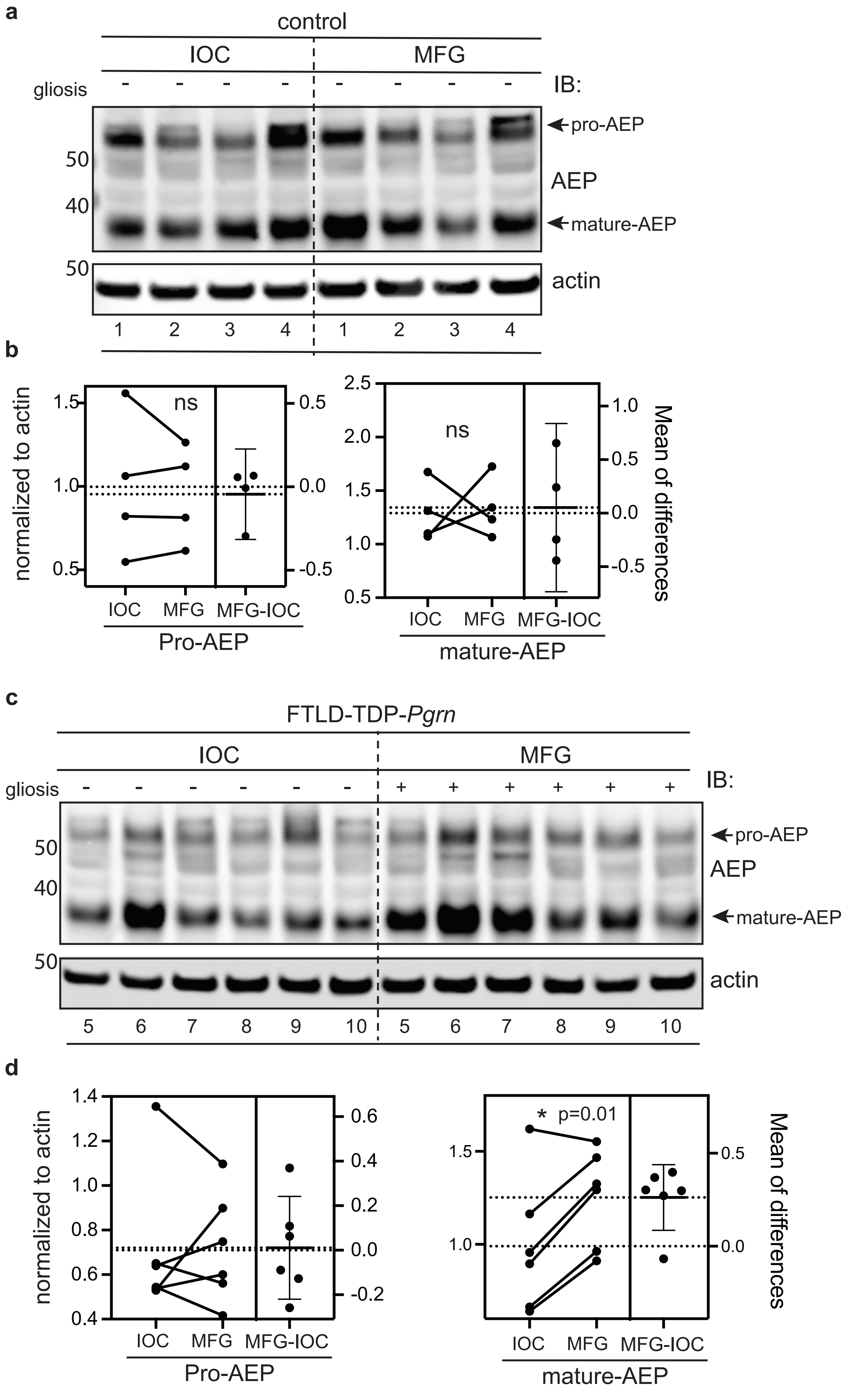

Supplement: Supplementary file 1 — Additional file 1 Fig. S1. Enzymes that do not cleave PGRN are biologically active. a, 1 μM of mature CTSD was incubated with 400 ng recombinant human PGRN or 400 ng BSA at the indicated pH. The incubation was stopped after 60 min or 16 h, and reaction contents were subjected to Coomassie staining (60 min) or silver staining (16 h) respectively. Bands correlate to proteins indicated on the right. BSA was used as a control for CTSD activity. At lower pH of 3.4, CTSD self-digest leading to a decreased level of mature CTSD compared to pH 4.5 [52]. b, FRET-based enzyme activity assay at pH 4.5 was performed on all enzymes that do not cleave PGRN. The values obtained were normalized to timepoint t = 0 and plotted relative to activity. All enzymes show activity against the optimized FRET peptides compared to substrate only control. Fig. S2. Antibody validation for individual granulin peptides. a, Dot blots confirming the specificity of each custom antibody towards the granulin peptide. 2.5 μg of each peptide was used with each antibody (11,000 dilution). Paragranulin, p-Gran; granulin G, Gran-G; granulin F, Gran-F; granulin B, Gran-B; granulin A, Gran-A; granulin C, Gran-C; granulin D, Gran-D; granulin E, Gran-E. b, Peptide blocking experiment to demonstrate antibody specificity. 400 ng of rhPGRN was incubated with 1uM CTSL for 20 min. The antibody was incubated with or without the respective blocking peptide (10 or 100-fold molar ratio of peptide to antibody) and then incubated with the membrane overnight. Increase in peptide concentrations show a decrease in the bands of each antibody tested. Fig. S3. Lysosomal proteases unable to digest PGRN in vitro. Full western blot images of the proteases that do not cleave PGRN. 1 μM of each enzyme was incubated with 400 ng of PGRN for 20 min. C-terminal Gran-E antibody or anti-PGRN (Invitrogen) antibody was used to assess the results of the in vitro assay. a, Cysteine proteases Cathepsins C (CTSC), H (CTSH), X (CTSX), O (CTSO) an [file 13024_2021_472_MOESM1_ESM.pdf]
